# Multidisciplinary studies with mutated HIV-1 capsid proteins reveal structural mechanisms of lattice stabilization

**DOI:** 10.1038/s41467-023-41197-7

**Published:** 2023-09-12

**Authors:** Anna T. Gres, Karen A. Kirby, William M. McFadden, Haijuan Du, Dandan Liu, Chaoyi Xu, Alexander J. Bryer, Juan R. Perilla, Jiong Shi, Christopher Aiken, Xiaofeng Fu, Peijun Zhang, Ashwanth C. Francis, Gregory B. Melikyan, Stefan G. Sarafianos

**Affiliations:** 1 C.S. Bond Life Sciences Center, University of Missouri, Columbia, MO USA; 2 Department of Chemistry, University of Missouri, Columbia, MO USA; 3 Center for ViroScience and Cure, Laboratory of Biochemical Pharmacology, Department of Pediatrics, Emory University School of Medicine, Atlanta, GA USA; 4 Children’s Healthcare of Atlanta, Atlanta, GA USA; 5 Department of Molecular Microbiology & Immunology, University of Missouri School of Medicine, Columbia, MO USA; 6 Department of Chemistry and Biochemistry, University of Delaware, Newark, DE USA; 7 Department of Physics & Beckman Institute, University of Illinois at Urbana-Champaign, Urbana, IL USA; 8 Department of Pathology, Immunology & Microbiology, Vanderbilt University Medical Center, Nashville, TN USA; 9 Department of Structural Biology, University of Pittsburgh, School of Medicine, Pittsburgh, PA USA; 10 Division of Structural Biology, University of Oxford, The Henry Wellcome Building for Genomic Medicine, Headington, Oxford UK; 11 Electron Bio-Imaging Centre, Diamond Light Sources, Harwell Science and Innovation Campus, Didcot, UK; 12 Department of Biological Science, Florida State University, Tallahassee, FL USA; 13 Division of Pediatric Infectious Diseases, Emory University School of Medicine, Atlanta, GA USA

**Keywords:** Viral proteins, X-ray crystallography, Retrovirus, Virus structures

## Abstract

HIV-1 capsid (CA) stability is important for viral replication. E45A and P38A mutations enhance and reduce core stability, thus impairing infectivity. Second-site mutations R132T and T216I rescue infectivity. Capsid lattice stability was studied by solving seven crystal structures (in native background), including P38A, P38A/T216I, E45A, E45A/R132T CA, using molecular dynamics simulations of lattices, cryo-electron microscopy of assemblies, time-resolved imaging of uncoating, biophysical and biochemical characterization of assembly and stability. We report pronounced and subtle, short- and long-range rearrangements: (1) A38 destabilized hexamers by loosening interactions between flanking CA protomers in P38A but not P38A/T216I structures. (2) Two E45A structures showed unexpected stabilizing CA_NTD_-CA_NTD_ inter-hexamer interactions, variable R18-ring pore sizes, and flipped N-terminal β-hairpin. (3) Altered conformations of E45A^a^ α9-helices compared to WT, E45A/R132T, WT_PF74_, WT_Nup153_, and WT_CPSF6_ decreased PF74, CPSF6, and Nup153 binding, and was reversed in E45A/R132T. (4) An environmentally sensitive electrostatic repulsion between E45 and D51 affected lattice stability, flexibility, ion and water permeabilities, electrostatics, and recognition of host factors.

## Abstract (web summary)

The effects of E45A or P38A capsid mutations on HIV core stability and infectivity are reversed by R132T or T216I. Here, authors used structural and biophysical methods to reveal short- and long-range rearrangements that explain stability changes.

## Introduction

During maturation of human immunodeficiency virus type 1 (HIV-1), capsid proteins (CA) assemble into a conical core (or capsid) surrounding the viral genome. Formation of a core with optimal stability is a strict requirement for efficient HIV-1 infection^1–3^. Following delivery into the cytoplasm, the HIV-1 core undergoes controlled disassembly (uncoating), which appears to be coordinated with productive reverse transcription and concealing the DNA product from immune surveillance of the target cell^4,5^.

The structure of CA and its effects on core stability are critical for uncoating, reverse transcription, nuclear entry, integration site selection, and assembly^2,3,6–12^. CA folds into two distinct domains connected by a linker: the N-terminal domain (CA_NTD_), composed of seven α-helices and a single β-hairpin, and the C-terminal domain (CA_CTD_), composed of a 3_10_-helix and four α-helices^6–8,13,14^. Purified HIV-1 CA can spontaneously assemble in vitro into tubes and cones that recapitulate the CA-CA interactions of authentic viral capsids^2,15–17^. The cores comprise ~250 CA hexamers and 12 CA pentamers^2,15–17^. A hexagonal lattice is the foundation of the mature capsid. It is stabilized by intra-hexamer (CA_NTD_-CA_NTD_ and CA_NTD_-CA_CTD_ contacts between six adjacent CAs in a hexamer), inter-hexamer (2-fold and 3-fold CA_CTD_-CA_CTD_ interactions between two or three adjacent CA hexamers, respectively), as well as intra-protomer (CA_NTD_-CA_CTD_ contacts in the CA monomer) interfaces^6–8,13,14,17–19^. To our knowledge, so far, there have been no reports of CA_NTD_-CA_NTD_ inter-hexamer interactions. It was previously shown that inositol hexaphosphate (IP6), stabilizes hexamers and promotes DNA synthesis, by binding at the six-fold symmetry axis of a CA hexamer, at a pocket composed of six R18 residues from each of the six CA monomers^20–30^.

Extensive research efforts have been invested over many years to determine the effect of residue changes on the structure and function of CA^1–3,31,32^. Among them, P38A and E45A did not cause obvious defects in assembly, maturation or the packaging of viral proteins, yet significantly reduced infectivity^3^. The infectivity reduction has been linked to altered core stability: P38A mutation destabilized viral cores, whereas E45A resulted in hyperstable cores^1^. Moreover, E45A decreased susceptibility to PF74^33–35^ and resistance to the restriction effects of CPSF6-358^36,37^ and TRIM-Nup153^37,38^. Resistance to PF74 is reversed in E45A/R132T^33–35^. Structures of cross-linkable HIV-1 CA complexes with PF74, CPSF6, or Nup153 have been available;^37–39^ however, mutations that were engineered to facilitate crystallization of hexameric CA (E45C, A14C, W184A, M185A), interfered with the comprehensive description of the inter-hexamer interactions. These primarily involve helices α9 at the 2-fold interface (α9_hex1 and α9_hex2), and helices α10 at the 3-fold interface (α10_hex1, α10_hex2, and α10_hex3). [Note: herein, we use the terms _hex1, _hex2, and _hex3 as descriptors for helices from hexamers 1, 2, and 3 that are engaged in *inter*-hexamer interactions; we refer to *intra*-hexamer interactions between helices in 3 neighboring subunits of the same hexamer using prime (΄), no prime, or double prime (΄΄), Supplementary Fig. 1]. To understand how CA binding to PF74, CPSF6 or Nup153 can be affected by changes at the 2-fold α9_hex1–α9_hex2 inter-hexamer interface imparted by the E45A mutation, we compared the structures of native WT CA in complex with peptides from CPSF6 or Nup153 solved herein, or with PF74 (previously described^18^). All structures have been solved in the native background, and thus encompass the authentic α9_hex1–α9_hex2 inter-hexamer interfaces.

In addition to the role of E45A and P38A we examined the role of respective second-site compensatory mutations, T216I and R132T, which have been selected by Yang et al.^33^. These mutations rescued the infectivity impairment caused by the original mutations without correction of the intrinsic capsid stability defect^33^ (data summarized in Supplementary Table 1). Structural elucidation of the effects of P38A, E45A, and R132T mutations has been limited to analyzing chemical shift changes by NMR spectroscopy using the purified N-terminal CA domain^33^. The exact structural effects of the mutations in the context of full-length CA and core assemblies remain unclear.

To address these knowledge gaps, we primarily focused our analysis on P38A, P38A/T216I, E45A, and E45A/R132T CA mutations. To understand how these affect access to the PF74/CPSF6/Nup153 binding pocket, we also solved structures of native WT with CPSF6 or Nup153 peptides and compared them to our previous WT, WT/PF74 structures in the native background. To characterize the effects of primary and compensatory mutations we assessed the assembly competence of CA mutants in vitro; conducted morphology studies using cryo-EM; analyzed core stability and uncoating using the CypA-DsRed live imaging assay; employed molecular dynamics (MD) simulations to assess in silico the effect of mutations on lattice thermal stability, ion and water permeability, flexibility, and electrostatics.

## Results and discussion

### Crystallographic analysis

We have crystallized and solved the structures of full-length CA bearing P38A, P38A/T216I, E45A, or E45A/R132T substitutions in the native, full-length WT CA background (Supplementary Fig. 2)^18^. Similar to CA WT, the crystal structures of CA mutants have been solved in space group P6 with one molecule per asymmetric unit (Supplementary Table 2). Flanking neighboring subunits of CA monomers are represented as CA΄ and CA΄΄ (left and right, respectively; nomenclature is described in Supplementary Fig. 1).

### X-ray structures of P38A and P38A/T216I CA

**Fig. 1:**
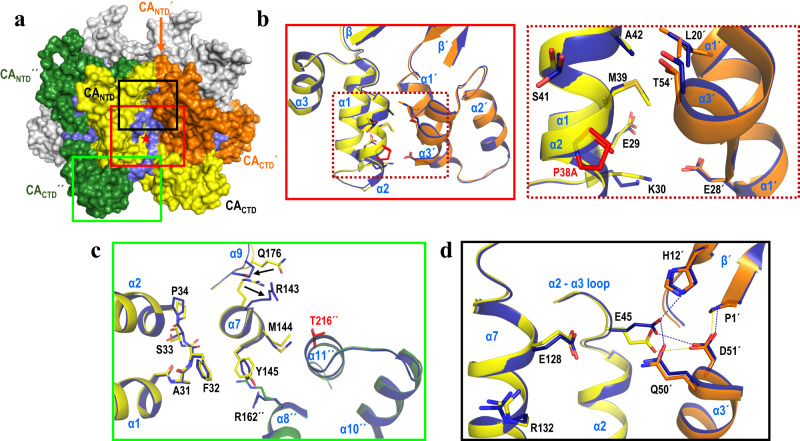
Structural changes associated with P38A mutation. **a** A CA hexamer is shown in surface view representation with three neighboring intra-hexamer CA monomers colored in orange (subunit ΄), yellow (subunit without prime symbol), and green (subunit ΄΄); the other three are shown in gray. P38A mutation site is marked with a red star in the yellow subunit. The P38A mutation affects regions in three subunits, shown in light blue surface view. **b**–**d** Superposition of WT (cartoon ribbons of three neighboring subunits colored in green, yellow, and orange) and P38A (in blue) CA. The mutation alters inter-hexamer CA_NTD_-CA_NTD_ **b**, **d** and CA_NTD_-CA_CTD_ interfaces **c**. Specific residues affected by P38A mutation (in red) are shown as sticks. For clarity, residues E35, V36, I37 are not shown. Dashed lines are shown between residues that are within 4 Å. Box colors in **b**–**d** correspond to the boxed regions in **a**. Dashed box in **b** is an insert of a region within the other box in **b**. For reference, the relative position of the revertant site, T216΄΄, not mutated here, is shown in red in **c**.

P38A CA: The structures of P38A (resolution 2.4 Å) and P38A/T216I (resolution 2.6 Å), are very similar to that of WT CA (RMSD 0.32 and 0.41 Å, respectively), demonstrating conservation of the overall global fold of the protein^6,18^. Residue 38 is located in the middle of the helix α2, which together with helices α1 and α3 form the 18-helix barrel at the center of the hexamer. Consistent with the previous structural assessment by NMR^33^ and compared to WT CA, P38A CA exhibits subtle changes that are dispersed over a wide region (Fig. 1). The affected residues are located proximal to the site of mutation in helices α1 (E29, K30), α2 (P34, E35, V36, I37, mutation site A38, M39, S41, A42), and in the neighboring intra-hexamer subunit (marked with ΄) in helices α1΄ (L20΄, E28΄), and in α3΄ (T54΄) (Fig. 1b). Moreover, the changes extend downstream from the site of mutation, altering the preceding loop between helices α1 and α2 (A31, F32, S33) (Fig. 1c). Further changes at the end of helix α7 (R143, M144, Y145) are mediated primarily via F32 in the affected loop, which in turn remodel the loop between helices α8 and α9 (Q176) (Fig. 1c). Furthermore, additional rearrangements are observed at the beginning of helix α8΄΄ (R162΄΄) in the other neighboring intra-hexamer subunit (marked with ΄΄) due to the changes in α7 (Fig. 1c). Finally, there are changes upstream from the mutation site that alter the network of interactions between E45 (in the loop between α2 and α3) within one subunit and residues of the neighboring subunit: P1΄ and H12΄ in the β΄-sheet), Q50΄ in helix α3΄, and D51΄ in helix α3΄ (Fig. 1d and Supplementary Fig. 3c). Notably, this network of interactions has been reported to potentially affect the β-hairpin conformation^40^.

Thus, the P38A mutation initiates a cascade of subtle changes that alter the network of *intra*-protomer (CA_NTD_-CA_CTD_), as well as *inter*-protomer (CA_NTD_-CA_NTD_΄ and CA_NTD_-CA_CTD_΄΄) interactions, thus altering the structure of three neighboring subunits within a hexamer (Fig. 1a). The structural changes cause a decrease in the calculated interface area (*IA*) at the inter-CA interfaces (Supplementary Table 3) that likely results in the observed broad destabilization and “loosening of the CA structure”.

Similar rearrangements are observed in the crystal structure of P38A/T216I, presumably due to the P38A mutation (Supplementary Fig. 3d and 4a–c). The compensatory T216I is in helix α11΄΄, proximal to helix α7 of the neighboring intra-hexamer subunit that is affected by the P38A mutation (Fig. 1c). T216I results in subtle rearrangements close to the mutation site in helix α11΄΄ (mutation site I216΄΄, Q219΄΄, V221΄΄), and also affects helix α10 (I201΄΄, L202΄΄, K203΄΄, A204΄΄) and the loop between α10 and α11 (G206΄΄, P207΄΄, G208΄΄) (Supplementary Fig. 4d). As a result, there is >200% increase in the *IA* at the 3-fold CA_CTD_-CA_CTD_ inter-hexamer interface, as compared to P38A. Moreover, a ~ 5% increase in the *IA* is observed at the other CA-CA interfaces (Supplementary Table 3). Notably, the T216I substitution instead of reversing the structural changes imparted by P38A, it induces subtle rearrangements that may lead to slight stabilization of the hexamers themselves as well as interactions between them, thus partially offsetting the destabilizing effect of P38A.

### Crystallographic analysis of E45A and E45A/R132T CA mutants

While E45A also crystallizes in space group P6 as P38A and P38A/T216I, surprisingly, we obtained data from crystals with two different unit cell dimensions: *a* = *b* = 87.6 Å, *c* = 56.5 Å (resolution 2.5 Å, labeled E45A^a^) and *a* = *b* = 92.5 Å, *c* = 57.8 Å (resolution 2.2 Å, labeled E45A^b^) (Supplementary Table 2). Both structures reveal the overall global fold to be essentially the same as in WT CA. Superpositions of E45A^a^ and E45A^b^ with WT CA revealed larger deviations of atomic positions (RMSD 1.44 and 0.81 Å) compared to P38A and P38A/T216I.

**Fig. 2:**
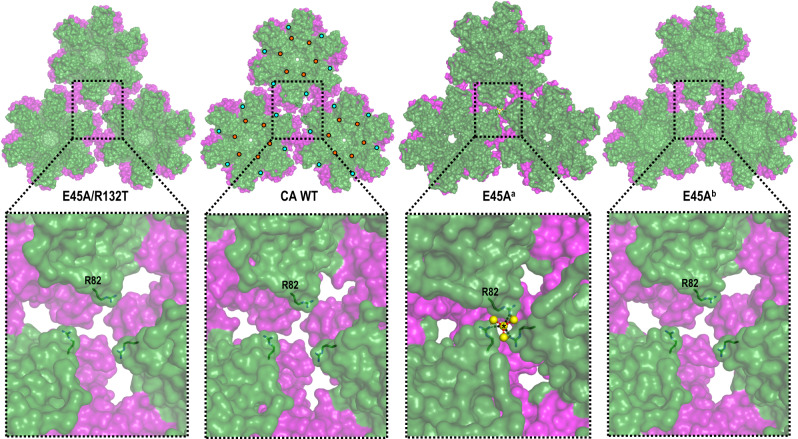
Arrangement of hexamers in the crystal structures of CA WT, E45A and E45A/R132T mutants. Neighboring hexamers in the lattices of CA WT, E45A^a^, E45A^b^, and E45A/R132T are shown in surface representation and also in enlarged views. CA_NTD_s are colored in green, CA_CTD_s in purple. R82 is labeled and shown as sticks. Residue 45 and 132 mutation sites are shown as orange and yellow circles, respectively. The 3-fold inter-hexamer interface interactions present in E45A^a^ and not in WT are: at the CA_CTD_-CA_CTD_ interface: I201/A204, K203/T216, K203/A217, K203/G220, A204/L205, P207/E212, P207/E213, and P207/T216 (Supplementary Table 6); at the CA_NTD_-CA_NTD_ interface: N21/A22, E35/V59, S41/H_2_O/Q50, A42/H_2_O/I15, L43/P17.

The ~5% difference in the unit cell dimensions between E45A^a^ and E45A^b^ is explained by the repositioning of the loop between helices α8 and α9 (~3 Å movement) in E45A^a^. This important movement primarily affects interactions at the 2-fold interface between neighboring hexamers; specifically, between helix α9 from one hexamer (α9_hex1) and the same α9 helix from the neighboring hexamer (α9_hex2). Of note, the adjoining 3_10__hex1 and 3_10__hex2 helices are also slightly rearranged in the E45A^a^ structure (Supplementary Fig. 5a and b). In addition, the changes are also translated to helices α10 and α11 that help form the 3-fold inter-hexamer interface (Supplementary Fig. 5a, b). As a result, the hexamers in E45A^a^ arrange more tightly than in WT CA or E45A^b^ (Fig. 2), forming extended interactions (Supplementary Table 6) at the 2-fold and 3-fold CA_CTD_-CA_CTD_ inter-hexamer interfaces that are distant from the location of mutation. Interface area calculations reveal that E45A^a^ has ~160% (726.2 vs. 453.2 Å^2^) and ~510% (236.4 vs. 46.3 Å^2^) more buried surface than WT CA at the 2-fold and 3-fold, respectively (Supplementary Table 3). Moreover, there are more inter-subunit interactions in E45A^a^ than in WT and in E45A^b^. Notably, a novel inter-hexamer interface is formed in E45A^a^ between N-terminal domains along the 3-fold symmetry axis (*IA* 23.9 Å^2^) mediated by R82 of the E45A^a^ CA_NTD_s (Fig. 2) and a H_2_O molecule (6 H_2_O per hexamer). To our knowledge, this interaction is absent in all other CA structures. Thus, the enhanced buried surface area and inter-subunit interactions observed in E45A^a^ may contribute to a “tighter” structure of E45A^a^, further stabilized by CA_NTD_-CA_NTD_ interactions at the 3-fold inter-hexamer interface, potentially contributing to the hyper-stabilization effect observed for HIV-1 E45A cores. Because there is considerably more buried surface area and inter-subunit interactions in E45A^a^ than in E45A^b^ we expect the former conformation to be more relevant for the stabilization effects of the E45A mutation.

The E45A mutation primarily remodels the neighboring network of interactions (Supplementary Fig. 3, Supplementary Table 4). This involves residues in the loop between helices α2 and α3 (S44, A45, G46), helix α7 (E128, K130, R132) of one subunit, as well as residues in helix α3΄ (Q50΄, D51΄) and β-hairpin (P1΄, H12΄) of the neighboring intra-hexamer subunit (Supplementary Fig. 3a, b, e, and f, Supplementary Table 4). As a result, there is a ~ 10% increase in the *IA* at the intra-hexamer interfaces of E45A^b^ (1,221.6 vs. 1,118.8 Å^2^). Additional subtle changes are observed at the inter-hexamer interfaces resulting in a ~ 10% (409.1 *vs*. 453.2 Å^2^) and ~40% (32.4 vs. 46.3 Å^2^) decrease in the *IA* at the 2-fold and 3-fold, respectively (Supplementary Table 3). Notably, this region was also altered by the P38A mutation (Supplementary Fig. 3a–d, Supplementary Table 6).

Similar changes are observed in the E45A/R132T (resolution 2.0 Å) structure (Supplementary Fig. 3g, Supplementary Table 4). The second-site mutation, R132T, located in helix α7 near E45A, partially restores extended water-mediated interactions impaired by E45A. Additionally, there is a decrease with respect to WT, in the *IA* at the intra- and inter-hexamer interfaces (Supplementary Table 3). This suggests that R132T may at least partially decrease the excess stability of the hexamers and hexagonal lattice imparted by E45A.

**Fig. 3:**
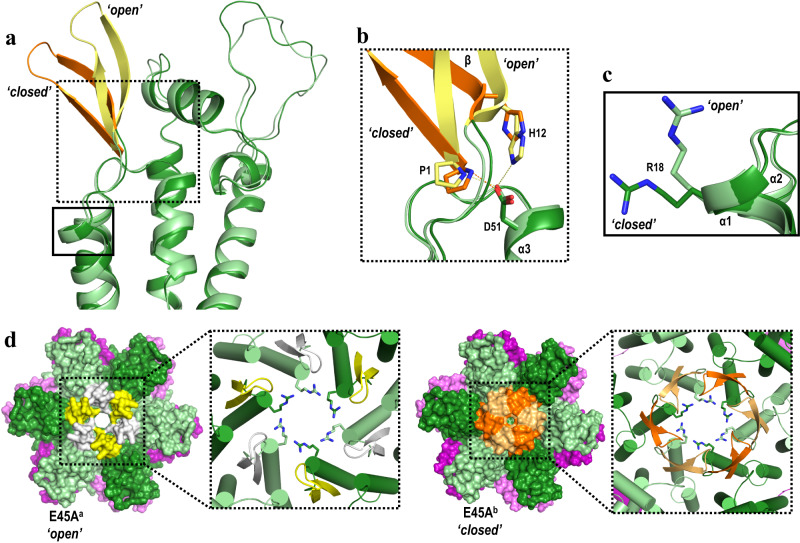
Changes in conformations of β-hairpin and R18 in E45A structures. **a** Superposition of N-terminal domains from E45A^a^ (CA_NTD_ in light green, β-hairpin in yellow) and E45A^b^ (CA_NTD_ in green, β-hairpin in orange). **b** Detailed view of the dashed boxed region in **a** shows that ‘open’ (yellow) and ‘closed’ (orange) β-hairpin conformations are the result of the hydrogen-bond network around P1, H12 and D51. **c** Detailed view of the solid boxed region in **a** shows that R18A toggles between ‘open’ and ‘closed’ states. **d** β-hairpin conformations dictate the presence of a pore at the 6-fold axis. E45A^a^ and E45A^b^ hexamers are shown in surface view representation with CA_NTD_s in light green and green, CA_CTD_s in light purple and purple. β-Hairpin in E45A^a^ structure is shown in white and yellow, while in E45A^b^ in light orange and orange. The boxed region shows a detailed view at the 6-fold axis in cartoon with R18 shown as sticks.

Surprisingly, comparison of the E45A^a^ and E45A^b^ structures with resolved β-hairpins revealed that they assume two distinct conformations that differ by ~11 Å (as measured by the displacement of the Q7 Cα): ‘open’ in the E45A^a^, and ‘closed’ in the E45A^b^ (Fig. 3a). Similarly, a comparison of available CA crystal structures^40^ showed that different β-hairpin conformations result from a pivoting movement of ~39° about the N-terminal proline (P1). In the E45A^b^ structure where the β-hairpin assumes ‘closed’ conformation, P1 forms a salt-bridge with D51^31^ (Fig. 3b). In the E45A^a^ where β-hairpin assumes ‘open’ conformation, D51 maintains interaction with P1 and now adds a second salt-bridge interaction with H12 (in yellow, Fig. 3b). Notably, six R18 residues in the middle of the ‘open’ E45A^a^ and ‘closed’ E45A^b^ hexamers, similarly adopt ‘open’ and ‘closed’ R18-ring conformations (Fig. 3c, d), resulting in pores with a diameter of ~11 Å and 5 Å, respectively. In the absence of biological data preferentially supporting one over the other E45A structures, it is possible that both conformations are present during infection.

Notably, the altered α9_hex1–α9_hex2 inter-hexamer conformation in E45A^a^ with respect to other CA proteins is restored to WT-like interactions in E45A/R132T (Supplementary Fig. 5a and b).

Thus, the combined effects of CA mutations (P38A and T216I or E45A and R132T) on core structure are highly complex, affecting not only the mutation sites, but also modulating inter- and intra-hexamer interactions.

### Comparison of WT/PF74, WT/CPSF6, WT/Nup153 with E45A^a^ and E45A/R132T crystal structures

The shared CA binding site targeted by PF74, CPSF6, and Nup153 is near α9΄ of a neighboring CA within the same hexamer (Supplementary Fig. 6a, Supplementary Tables 5 and 6). At the same time, α9 forms the inter-hexamer 2-fold interface by interacting with α9 from a neighboring hexamer (α9_hex1–α9_hex2; Supplementary Fig. 5a, b, b, and d). Because in E45A^a^ the α9_hex1–α9_hex2 inter-hexamer interaction is changed with respect to WT but restored to WT-like in E45A/R132T (Supplementary Fig. 5a and b), we hypothesized that the conformational variability of the α9_hex1–α9_hex2 inter-hexamer region affects the phenotypic differences in ligand binding at the PF74/CPSF6/Nup153 pocket of various Cas. To help address this hypothesis we also solved structures of native WT CA complexes with CPSF6 (WT_CPSF6_) or Nup153 (WT_Nup153_) peptides (Supplementary Table 2 and Supplementary Fig. 6) at 2.5 and 2.4 Å resolution, and analyzed them together with E45A^a^ and our previous native WT CA, WT_CA_, and WT_PF74_ structures^18^, thus enabling structural comparisons in the context of native CAs bound to PF74/CPSF6/Nup153 ligands. The reason we could not rely on information on α9_hex1–α9_hex2 from previously solved structures of cross-linked CA in complex with CPSF6 (CA_XL-CPSF6_) or Nup153 (CA_XL-Nup153_), is that mutations W184A and M185A at the α9 helix severely affected the inter-hexamer region:^37,39^ specifically, we see significant differences between WT_CPSF6_ and CA_XL-CPSF6_ or between WT_Nup153_ and CA_XL-Nup153_ at the 2-fold (Supplementary Fig. 6c) regions. Comparison of WT_CA_ to WT_CPSF6_ or WT_Nup153_ revealed some minor structural differences at the 2-fold (Supplementary Fig. 6d) and 3-fold (Supplementary Fig. 6e) regions, and binding of the CPSF6 or Nup153 to native WT_CA_ CA (WT_CPSF6_ or WT_Nup153_) or the cross-linked CA_XL_ (CA_XL-CPSF6_ or CA_XL-Nup153_) was essentially the same.

Comparison of the E45A^a^, E45A/R132T, WT_CA_, WT_PF74_, WT_CPSF6_, and WT_Nup153_ revealed that repositioning of the α9 helix in E45A^a^ may lead to steric interference that could affect access to the PF74/CPSF6/Nup153 pocket (Supplementary Fig. 7). In turn, the R132T secondary mutation in E45A/R132T appears to restore the local structure to WT-like conformation, likely enabling unfettered access to the PF74/CPSF6/Nup153 pocket (Supplementary Fig. 7). Lenacapavir (LEN), a long-acting capsid-targeting antiviral developed by Gilead^41^ and recently approved for anti-HIV therapy as Sunleca, also binds at this pocket^42^. Therefore, we expect that the structural changes in the α9-helix will prevent binding of these antivirals. Of note, the recently published clinical reports on LEN resistance mutations do not include E45A, likely because this mutation structurally disrupts the pocket where host factors CPSF6 and Nup153 need to be accommodated for the virus to be able to replicate.

### Morphology studies using cryo-EM

It is known that CA is capable of different assembly pathways in vitro depending on the protein concentration, pH, or ionic strength^43–45^. The sensitivity of the higher-order structures to such factors indicates that polar interactions on the multimer surface regulate their formation^43^. Previous studies of the assembly properties of CA mutants by transmission electron microscopy (TEM) revealed that P38A and E45A assembled with efficiencies similar to that of the CA WT^2^ at protein concentrations of 5, 10, and 15 mg/ml.

To evaluate the effects of compensatory mutations on the assembly competence of CA mutants, we incubated CA at high salt and analyzed cylinder formation in cryo-electron micrographs (cryo-EM). Under these conditions, P38A CA does not assemble into tubular structures; however, tubular assembly was rescued by T216I (Supplementary Fig. 8a, b). Consistent with the structural diversity observed in the E45A^a^ and E45A^b^ structures, E45A CA assembles into a mixture of short tubes and cones (Supplementary Fig. 8a, b). As in P38A/T216I, R132T, reverts the E45A assembly morphology to that of WT CA long tubes. These results are consistent with the pelleting assay that allows to evaluate assembly efficiency of CA proteins (Supplementary Fig. 8c). The E45A CA assembles more efficiently comparted to the CA WT and other CA mutants, while the assembly of P38A CA is largely impaired. These observations are consistent with the characterization of CA mutants P38A as unstable and E45A as hyperstable^1^. Interestingly, under the conditions of this assay, compensatory mutations T216I and R132T, reverted the effect of the primary mutation on the assembly competence of mutant proteins.

### Evaluation of core stability using CypA-DsRed loss assay

Biochemical studies have shown that detergent-treated purified capsids tend to disassemble in a temperature-dependent manner, and mutations in CA can strongly modulate core stability^1^. Consistent with our data (Supplementary Fig. 9a–c), intact cores could be recovered from WT, but not from P38A virions, presumably due to their reduced stability. Cores from E45A CA yielded greater quantities, and were found to disassemble more slowly than WT CA-containing cores, when heated at 37 °C^1^. Notably, cores recovered from P38A/T216I and E45A/R132T did not behave very differently from P38A and E45A^33^.

We have used virus-imaging to evaluate the effect of mutations on the stability of cores. This strategy enables visualization of HIV-1 uncoating using a fluorescently-tagged oligomeric form of a capsid-binding host protein, cyclophilin A (CypA-DsRed), which is specifically packaged into virions through high-avidity binding to CA^46^. Single virus imaging revealed that CypA-DsRed remained associated with cores after permeabilization/removal of the viral membrane and that CypA-DsRed and CA were lost concomitantly from the cores in vitro and in living cells^46^. The rate of loss was modulated by the core stability and was accelerated upon the initiation of reverse transcription.

To evaluate the effects of P38A, P38A/T216I, E45A, and E45A/R132T CA mutations on uncoating and core stability, we employed the CypA-DsRed loss assay. CypA-DsRed was lost from the permeabilized E45A particles much slower than from WT particles (Supplementary Fig. 9a, b. Addition of R132T resulted in a decreased stability for the E45A/R132T cores, albeit still higher than that of WT, especially at later time points (Supplementary Fig. 9b). The vast majority of CypA-DsRed puncta disappeared in less than 5 minutes after membrane permeabilization of the P38A viruses (Supplementary Fig. 9a, c). The P38A/T216I cores became slightly more stable than P38A at later time points. Hence, consistent with previous reports^33,47–49^, the second-site suppressor mutations do not fully correct the respective stability defects imposed by E45A or P38A.

### Molecular dynamics simulations

We also used MD simulations to probe the following correlates of structural stability: in silico thermal stability, the ion and water permeabilities, structure flexibility, electrostatics, as well as inter- and intra-hexamer interactions in WT and mutant lattices.

**Fig. 4:**
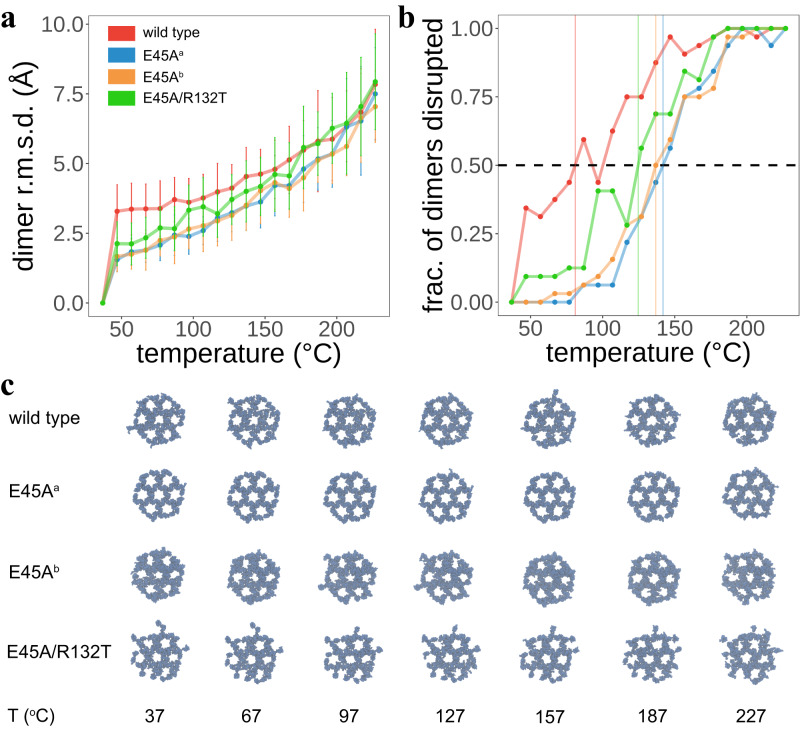
In silico thermal stability assay of WT and mutant CA lattices. **a** Dimer interface RMSD analysis for 2 × 3 × 3 hexameric CA lattices. Four constructs, WT, E45A^a^, E45A^b^, and E45A/R132T, were subjected to tempering simulations. Temperatures employed for NPT simulations were between 310 K and 500 K with a stride of 10 K. The RMSD is presented as the mean and standard deviation, of all dimers in each lattice (*n* = 32 dimer interfaces from each 2 × 3 × 3 hexameric lattice simulation), between backbone atoms comprising the interface. The reference structures were taken as the respective dimer interface simulated at 310 K. This reference point is included in the plot for clarity. **b** T_m_ profiles for all CA lattice constructs, based on the RMSD data shown in **a**. Based on the RMSD analysis, we consider a dimer interface to be disrupted if it deviates from its 310 K reference structure with a heavy-atom RMSD > 3.5 Å. T_m_ is the temperature where half of the dimer interfaces in the lattice are disrupted. This is visible as the point of intersection between the traces and the horizontal dashed line at a y-axis position of 0.5. For WT, this is 81.0 °C; for E45A^a^ and E45A^b^, these are 141.8 and 136.9 °C, respectively; for E45A/R132T, this is 124.6 °C. **c** Snapshots of 1 × 3 × 3 lattice CTDs taken at select intervals across the temperature range sampled, providing a qualitative view of the lattice stability. E45A^a^ is robust even at the highest simulation temperatures. Snapshots of NTDs and CTDs for every temperature are shown in Supplementary Fig. 10.

To assess the stability of the WT CA lattice compared to the hyper stable E45A and compensatory R132T mutant lattices, we performed tempering MD simulations to derive lattice melting temperature profiles (Fig. 4). As outlined in Supplementary Methods, we subjected each CA lattice construct to constant-pressure constant-temperature (NPT) simulations across a temperature range (310-500 K) with a stride of 10 K. We then analyzed the dimerization domains of the CA lattice CTDs to assess stability, using RMSD of the temperature replicas against the reference simulated at 310 K. This RMSD was then utilized to determine whether a dimer interface was disrupted; disruption was considered as a dimer interface heavy-atom RMSD > 3.5 Å from its 310 K reference. All computed RMSDs are shown in Fig. 4a.

In Fig. 4b, we show the fraction of disrupted dimers versus temperature for each CA construct; we consider the simulation temperature where half of all dimer interfaces are disrupted as the T_m_. Remarkably, both E45A^a^ and E45A^b^ lattices are more stable than the WT and E45A/ R132T lattices. WT CA is the least stable, with a T_m_ of 81.0 °C. E45A^a^ and E45A^b^ lattices are the most stable (T_m_ = 141.8 and 136.9 °C, respectively). For E45A/R132T CA, there is a loss in stability (T_m_ = 124.6 °C). Our simulations corroborate that E45A significantly stabilizes the lattice, likely through abundant resulting CTD contacts. Further, addition of R132T confers a decrease in stability, matching what is observed experimentally for E45A/R132T CA. Figure 4c shows snapshots of the lattice CTDs at some of the simulated temperatures (Supplementary Fig. 10 shows both NTD and CTD snapshots for all systems across every simulated temperature).

To mimic the interior and exterior of the capsid, we built a model of two 3 × 3 planar hexamer lattices in a mirrored configuration (Supplementary Fig. 11, see Supplementary Methods for additional details). The middle water layer, enclosed by the CA_NTD_s of the hexamers, represents the exterior environment of the HIV-1 capsid (Supplementary Fig. 12). Water molecules and ions in the model, were only able to exchange from/to the exterior to/from the interior of the capsid by crossing the hexamer layers. Using this model, we calculated the transfer rates of ions and water molecules in the case of WT and mutated CA hexamers (Supplementary Fig. 13). For each hexamer, the inwards and outwards transfer rates of each species were found to be similar. This suggests that ions and water are in thermal equilibrium between the inside and outside of the hexamer lattices. Interestingly, in all cases the transfer rate of chloride is much higher (at least two-fold) than the transfer rate of sodium (Supplementary Fig. 13a vs. b). In particular, the transfer rate of chloride through WT CA hexamers is 2.72 ± 0.33 and 2.77 ± 0.31 ions per ns, inwards and outwards, respectively (Supplementary Fig. 13a), whereas for sodium, the transfer rates through WT CA hexamers are 0.65 ± 0.16 (inwards) and 0.69 ± 0.26 (outwards) ions per ns (Supplementary Fig. 13b). Comparison of the ion transfer rates of mutated vs. WT CA hexamers, suggests that in general, mutations reduce the ability of the hexamers to transport ions to variable extent, except in the second-site suppressor mutant E45A/R132T and E45A^b^ (Supplementary Fig. 13a). Interestingly, the E45A^a^ CA hexamer has the lowest Na^+^ and water permeability among mutant hexamers. Nonetheless, the ability of the E45A^a^ CA hexamers to translocate ions and water is recovered by the introduction of the second-site mutation E45A/R132T. Notably, the P38A mutant reduces the anion permeability of the protein, while introduction of T216I in P38A/T216I slightly increases the chloride ion permeability (Supplementary Fig. 13a).

CA flexibility was assessed by means of the root mean squared fluctuation (RMSF) calculated using VMD, where larger fluctuations represent more flexible regions of the protein. Comparison of the RMSF of Cα for WT and mutant CA hexamers in Supplementary Fig. 14 shows the disordered or mobile regions (coils, turns) to have higher RMSF values than the helices. Moreover, comparison of the E45A^a^ hexamer with WT and E45A/R132T mutant hexamers suggests that E45A^a^ CA has slightly lower mobility (smaller RMSF values) in the C-terminal domains (Supplementary Fig. 14a). Among the three E45A^a^, E45A^b^, and E45A/R132T mutants, the generally slightly smaller RMSF value for E45A^a^ suggests that it is more rigid in the CA_CTD_ region. Similarly, comparison of the RMSF between CA WT, P38A, and P38A/T216I, indicates similar flexibilities among them, except the N- and C-terminus (Supplementary Fig. 14b). Moreover, the structural changes of WT and mutant hexamers (Supplementary Fig. 14c) also show that E45A mutations have smaller RMSDs with respect to their crystal structures. Hence, the mutations appear to have insignificant effects on the flexibility of CA, except E45A that reduce the flexibility of CA, especially in the C-terminal domains.

Electrostatic maps were calculated for single hexamer (Supplementary Fig. 15, Supplementary Fig. 16) and 3 × 3 lattices (Supplementary Fig. 17) of CA WT and mutant proteins. The electrostatic maps revealed two charged areas in the surface of the capsid. In particular, all hexamers present a highly positively charged area located in the central pore. Similarly, negative charges are localized in the boundary of hexamer-hexamer interfaces, particularly the two-fold and three-fold interfaces. The observed electrostatic changes at regions known to affect core stability and are thus consistent with the lattice stability changes calculated by the MD simulations. Electrostatic and Van der Waals (VDW) interaction energies between individual hexamers and their six surrounding neighbors were calculated for WT and mutant CA to evaluate the strength of the interactions between the respective hexamers. Overall, the electrostatic energy does not exhibit significant differences among hexamers from various proteins (difference <3%). Importantly, the two E45A single mutants display markedly stronger VDW *inter*-hexamer interactions than the WT and other mutants do (Supplementary Fig. 18). In contrast, addition of R132T to E45A reverses the E45A changes, leading to similar inter-hexamer VDW interaction energies for WT and E45A/R132T.

### Implications for the structural mechanism of HIV-1 capsid stability and uncoating

Uncoating, or disassembly of the viral capsid, is a critical, yet poorly understood step of the HIV-1 life cycle. In the present work, we observed that T216I and R132T mutations can at least partially correct in vitro assembly defects imposed by P38A and E45A, respectively, without fully correcting the altered core stability (disassembly in Supplementary Fig. 9b, c) imposed by the original mutations. As suggested previously^33^, one possible explanation for this discrepancy could be that the available assays, even though improved over time, may be limited in their ability to detect subtle changes in capsid stability. Alternatively, the suppressor mutations may alter interactions with host factors that participate in HIV-1 uncoating in target cells.

The core stability is intrinsically connected to the strength of the interactions between the building blocks (mostly hexamers but also few pentamers) and between the monomers within each building block, with the latter having larger impact on the CA stability^50^. While crystal structures represent a single averaged conformation, they relate to the diverse CA subunits in an actual fullerene cone capsid as they likely recapitulate part of the large spectrum of similar conformations. The MD simulation stability data at the lattice level strengthen the relevance of these studies, as they show that a flat lattice recapitulates the defects on stability.

Our structural analysis of P38A, P38A/T216I, E45A, and E45A/R132T CA proteins revealed short- and long-range rearrangements in HIV-1 CA. The structure of E45A^a^ exhibited notable changes with respect to WT, E45A^b^ and E45A/R132T structures: changes in CA_NTD_-CA_NTD_ inter-hexamer interactions in Fig. 2; changes in CA_CTD_-CA_CTD_ (α9_hex1 with α9_hex2) inter-hexamer interactions, in Supplementary Fig. 5a, b; beta-hairpin region and ring-of-six-R18 pore size changes in Fig. 3; and changes in intra-hexamer interactions (α3΄ with α2 and with β΄) in Supplementary Fig. 3.

The P38A mutation, by itself, or present together with T216I, initiates a cascade of subtle changes that result in remodeling of the CA-CA interaction network in a hexamer (Fig. 1). Notably, in the P38A structure (but not in P38A/T216I), these changes also affect the network of interactions between E45 (which is at the top of the α2 helix where 38 is present) of one subunit and residues of the neighboring hexamer. The second-site mutation T216I is at the 3-fold inter-hexamer interface; it induces further rearrangements that may lead to stabilization of interactions between hexamers, thereby partially offsetting the effect of P38A (Supplementary Fig. 4). On the other hand, E45A primarily remodels the network of polar and water-mediated interactions proximal to the site of mutation. R132T is near E45A and partially restores impaired water-mediated interactions (Supplementary Fig. 3g). Notably, second-site mutation-induced reversal of altered interactions around residue 45 are observed not only in E45A/R132T *vs*. E45A structures, but also in P38A/T216I *vs*. P38A structures. Hence, a key conclusion is that residue 45 is involved in capsid stability directly, as well as indirectly.

In terms of electrostatic changes, the E45A, and to a lesser extent, the P38A mutations alter the surface electrostatic potential in CA hexamers. Specifically, while E45 in WT CA is engaged in both attractive and repulsive ionic interactions with D51 from the neighboring subunit (Supplementary Fig. 3b, Supplementary Table 5), the E45A mutation relieves local electrostatic repulsion (Supplementary Fig. 3e, f, 16b, c, Supplementary Table 5), thus stabilizing the E45A CA hexamer. In addition, both E45A^a^ and E45A^b^ structures have variable interactions of the beta hairpin base with D51, also resulting in local electrostatic changes (Fig. 3b and Supplementary Figs. 3e, f, 16b, c). In P38A the interactions around E45 are altered, leading to an increase in the electrostatic repulsion between E45 and D51, and destabilization of the P38A CA hexamer (Fig. 1d, Supplementary Figs. 3c and 15b, Supplementary Table 5). Compensatory mutations T216I and R132T, respectively, may affect the charge distribution of CA (Supplementary Figs. 3d, g, 15c, and 16d). Those local changes in surface electrostatic potential may help explain differences in susceptibility to PF74 and interactions with host factors, including CPSF6, Nup153, TRIM5, CypA, TNPO3, and nucleoporins from the nuclear pore complex (Supplementary Table 1). Most importantly, the unique interactions between the α9_hex1 and α9_hex2 helices from neighboring hexamers in the E45A^a^ structure (Supplementary Fig. 5a, b), directly affect access to the common binding site of PF74, Nup153, and CPSF6 (Supplementary Fig. 7a, b). This decreased accessibility is not present in E45A/R132T, as the structure of this region is restored to a WT-like conformation (Supplementary Fig. 5b). Collectively, our data shed light on how the changes in α9_hex1 and α9_hex2 helices in E45A^a^ affect binding of PF74^33–35^, decrease in restriction by CPSF6-358^36,37^, and by TRIM-Nup153^37,38^ and also show how E45A/R132T may reverse such effects as it restores the structure of this region to a WT-like conformation (Supplementary Fig. 5b).

The T216I mutation has been shown to reverse the impaired ability of the P38A mutant to abrogate TRIMCyp- and TRIM5α-mediated restriction of HIV-1 (Supplementary Table 1)^33^. It has been shown that TRIM5α binding involves multiple capsid molecules along the 2-fold and 3-fold inter-hexamer interfaces^51,52^. Thus, our observed structural changes at the 3-fold interface in P38A/T216I may explain the rescue of the ability to abrogate TRIM5α restriction. Alternatively, T216I may simply restore capsid stability and thus the ability to deplete TRIM5α. Recent statistical analysis implicates the CypA binding loop region in binding of CA TRIM5α^53^. As this loop is involved in crystal contacts, any changes between mutant CA structures at this location would be difficult to interpret.

Unexpectedly, E45A CA crystallized in two different forms under very similar crystallization conditions. While the overall fold of E45A in both structures is essentially the same, their inter- and intra-hexamer interactions differ; the E45A^a^ structure appears to be stabilized by a novel 3-fold inter-hexamer interface formed between N-terminal domains of the E45A CA (residue R82 in Fig. 2). To our knowledge, this is the first report of C_NTD_-C_NTD_ inter-hexamer interactions that may be a major factor in the increased stability of the E45A capsid. The differences in intra-hexamer interactions are caused by changes at multiple sites. Loss of interactions between E45 and D51 from the neighboring subunit (Supplementary Fig. 3b vs. e, and f, Supplementary Tables 4 and 5) enables D51 to form H-bonds with either P1 or H12 residue, while remaining virtually unmoved (Fig. 3b). The two different conformations (E45A^a^ and E45A^b^) are likely the result of differences in the protonation state of H12 (crystals were set at pH ~6.5, near the pKa of H12), which in turn affected the H12-D51 interactions (Supplementary Fig. 3e, f). This is consistent with the proposed effect of pH on the conformation of the β-hairpin^40^. Remarkably, the differences in H12-D51 interactions directly result in significant changes at the top of the pore at the 6-fold symmetry axis, where the β-hairpin was observed in an “open” conformation in E45A^a^ and a “closed” conformation in E45A^b^ (Fig. 3). Likely connected to these rearrangements at the top of the pore, we also observe changes deeper in that channel: specifically, the six R18 side chains are in the “up” conformation in E45A^a^ where the β-hairpin is “open”, and in the “down” conformation in E45A^b^ where the β-hairpin is “closed” (Fig. 3c, d). The ring of R18s forms a pore of variable size ( ~ 11 Å diameter in E45A^a^), which has been proposed to facilitate transit of negatively charged small molecules (including deoxynucleoside triphosphates, dNTPs) to the interior of HIV-1 E45A capsid^40^. The two solved E45A structures confirm maximum pore flexibility under near physiological conditions. Of note, the E45A structures can only be structurally studied with our crystallographic system of native CA, as the cross-linkable crystallographic system^6,40^ relies on an engineered disulfide bond between E45C and A14C.

It has been previously observed that E45A cores appear to be more permeable to fluorescent dyes than WT cores^54^. Moreover, E45A HIV-1 exhibited a rapid viral RNA decay profile and a more rapid accumulation of early reverse transcripts. As a result, it was concluded that the E45A capsid dissociated early after infection^54^. Our data provide evidence that enlarged pores can exist in the intact E45A capsid, allowing an enhanced influx of dyes and dNTPs, which may explain the observed phenotypes.

It has been proposed that the highly hydrated character of the CA is compatible with the quasi-equivalent switching mechanism, because water molecules should be particularly adept at repositioning to accommodate altered orientations in hydrogen bonding and side chain packing geometries^6,8^. Interestingly, the hyperstable E45A has a significantly different hydration layer than the unstable P38A, consistent with the hypothesis that structured water molecules may contribute to the stabilization of capsid^18^.

We propose that the intra-hexamer electrostatic repulsion between E45 and D51 from neighboring CA monomers is a functionally important interaction. These residues are forced into proximity within the hexamer structure and they are also interacting with one or both ends of the β-hairpin (P1 and H12, Supplementary Fig. 3). Hence, this network of interactions can provide an environmentally-sensitive switch that can affect the conformation of the β-hairpin, the presence of the pore, and also the stability of the core and its disassembly (or uncoating) of the viral capsid. This mechanism is additionally supported by site-directed mutagenesis data, as mutating the carboxylate group of D51 to the corresponding amide (N51) results in capsids that exhibit significantly increased stability compared to CA WT^55^. Moreover, D51N formed long tubular structures in vitro comparable to CA WT, both in terms of external diameter and length of the tubes^56^.

Collectively, the structures support the hypothesis that CA plasticity is a key factor for its stability and interactions with antivirals and host factors. The possibility of capsid stability regulation through changes in pH, dNTP recruitment and DNA synthesis, provides a model whereby DNA synthesis is coordinated with uncoating to cloak the viral genome from cytoplasmic DNA sensing.

## Methods

### Design, expression and purification of CA mutants

WT, P38A, P38A/T216I, E45A, E45A/R132T CA proteins were cloned in a pET11a construct^18^. Mutations were introduced using overlap extension PCR cloning and verified by DNA sequencing. WT and mutant P38A, P38A/T216I, E45A, E45A/R132T CA proteins were expressed and purified as previously described^18,57^.

### Crystallization of CA mutants

Crystals of WT P38A, P38A/T216I, E45A, E45A/R132T CA grew at 18 °C in hanging drops, containing 2–5 mg/ml of protein, 2–14% PEG 3350, 2–6% glycerol, sodium iodide, and sodium cacodylate. Hexagonal plate-like crystals appeared after 5 days and crystal growth was completed in over 2 weeks. Crystals were briefly soaked in 20% glycerol or paraffin oil before cryo-cooling in liquid nitrogen. For the WT_CPSF6_ and WT_Nup153_ complexes, peptides (CPSF6_313–324_ with sequence PVLFPGQPFGQP and Nup153_1407–1423_ with sequence TNNSPSGVFTFGANSST) were soaked into unliganded WT crystals for approximately 24 h.

### Data collection and structure determination

Data were collected on a MAR CCD (23-ID-B), Dectris Eiger-16m (23-ID-B) or Pilatus3 6 M (23-ID-D) detectors at the Advanced Photon Source, Sector 23, and on a CMOS detector at Advanced Light Source (ALS) beamline 4.2.2, Lawrence Berkeley National Laboratory. Datasets were collected and processed using XDS^58^. The data were examined for the presence of systematic absences, however, no characteristic patterns were observed. Thus, the crystals were indexed in hexagonal space group P6 with one CA molecule in the asymmetric unit. No twinning was present, as determined by either POINTLESS (version 1.10.21)^59^ or XTRIAGE (version 1.11.1-2575_1692)^60^. Space group and twinning were also verified by ZANUDA^61^. The phase problem of CA was solved either using single-wavelength anomalous diffraction (SAD) or molecular replacement, with the native CA (PDB ID: 4XFX) as the starting model. For SAD, substructure solution, phasing, density modification, model building, and refinement were carried out using SHELX C/D, SOLOMON, PARROT, BUCCANEER and REFMAC in CRANK-2 (version 2.0.111)^61^. For molecular replacement, initial phases were solved via PHASER (version 2.7.17)^61^. Several rounds of iterative model building and refinement were carried out using Coot (version 0.8.8)^62^ and PHENIX (version 1.11.1-2575_1692)^60^, REFMAC (version 5.8.0155)^61,63^, or PDBREDO (version 6.24; https://pdb-redo.eu/), respectively. Structure validation of final models was performed with MOLPROBITY (version 4.5.2; http://molprobity.biochem.duke.edu/). Accessible and buried surface area were calculated using PISA (version 1.5.0)^61^. The figures showing structural information were generated in PyMOL (version 1.7.6.7; http://www.pymol.org/). Coordinates and structure factors have been deposited in the RCSB Protein Data Bank (PDB; see additional details in the Data Availability statement). Data collection and refinement statistics are provided in Supplementary Table 2. Representations of the asymmetric unit, biological assembly, and electron density maps of regions of interest can be found in Supplementary Fig. 19.

### Pelleting assay

CA WT and mutants (P38A, P38A/T216I, E45A, and E45A/R132T) were assembled at 2 mg/ml (80 µM) in buffer containing 1 M NaCl and 50 mM Tris-HCl pH 8.0 at 37 °C for 1 h. A total of 5 µl samples were withdrawn from the reaction mixtures and immediately used for cryo-EM analysis. The remaining sample was pelleted at 21,000×g for 30 min at 4 °C. Supernatants (S) and pellets (P) were mixed with 4 × LDS loading buffer (Invitrogen) supplemented with 10 mM dithiothreitol (DTT), without boiling, resolved on a 10% SDS-PAGE gel and stained with Coomassie Blue. Experiments were performed as three biological replicates, with one representative gel shown in Supplementary Fig. 8c.

### Morphology studies of CA mutants

The fresh assembled samples (4 μl) were applied to the carbon side of a glow discharged perforated Quantifoil grid (Quantifoil Micro Tools, Jena, Germany). The grids were then manually blotted with a filter paper from backside to remove the excess fluid, and plunge-frozen in liquid ethane using a home-made gravity plunger. For cryo-EM imaging, the frozen grids were loaded into a cryo-holder (Gatan Inc., Pleasanton, CA), inserted into a Tecnai F20 transmission electron microscope (FEI, Inc., Hillsboro, OR) and imaged with a 4k×4k charge-coupled device camera (Gatan). Low dose ( ~ 20 e^−^/Å^2^) projection images were recorded at a nominal magnification of 50,000 × with a pixel size of 2.26 Å and underfocus values ranging from 3.0 to 5.0 µm. The low magnification images were recorded at the magnification 5,000 ×. Experiments were performed as three biological replicates, with representative images shown in Supplementary Fig. 8a, b.

### CypA-DsRed loss assay

HIV-1 viruses bearing P38A, P38A/T216I, E45A, and E45A/R132T mutations were produced in 293 T cells (obtained from and validated by ATCC, Manassas, VA) by incorporating INsfGFP and CypA-DsRed^46^. Viruses were bound to poly-l-lysine treated coverglass and viral membrane was permeabilized with 100 µg/ml saponin for 1 min followed by 1× wash with Dulbecco’s phosphate-buffered saline (dPBS). The solution was replaced with 200 µl dPBS, and four fields of view were imaged at room temperature. The total numbers of INsfGFP spots and CypA-DsRed spots as a function of time were determined for each condition. HIV-1 core (CypA-DsRed) retention was calculated for the first time point for each CA mutant by using the respective INsfGFP-signal (pre-/post-saponin) as reference. The core stability was determined by plotting the loss of CypA-DsRed spots over-time, normalized to the initial number of spots. The number of INsfGFP spots remained constant and served as a reference marker. Cyclosporine A (CsA) 5 μM was added at 22 min after virus permeabilization. Immature particles that retained CypA-DsRed and failed to respond to CsA treatment were excluded from analysis. Plots in Supplementary Fig. 9 are means and standard errors from 4 independent experiments; for each experiment,

### Molecular modeling

#### Model building

The structures of mutant CA hexamers, namely E45A^b^, E45A^a^, E45A/R132T, P38A, and P38A/T216I were used as the starting point for all mutant simulations in the present study; missing residues were added using Modeller^64^. For simulations of CA WT, a previously reported structure of the hexamer was employed (PDB ID: 4XFX). To mimic the interior and exterior of the capsid, two 3 ×3 planar hexamer lattices were built in a mirrored configuration (Supplementary Fig. 11). The facing lattices were placed at least 4 nm away from each other to avoid interactions between the two protein layers. The middle water layer, enclosed by the CA_NTD_s of the hexamers, represents the exterior environment of the HIV-1 capsid (Supplementary Fig. 12). Conversely, the other two layers of water represent the interior of the capsid and are connected together due to the periodicity of the simulation along the z-axis. It is worth noting that water molecules and ions in the model could only exchange between the exterior and the interior of the capsid by crossing the hexamer layers.

The resulting lattices were solvated using the TIP3P water model employing the Solvate 1.5 plugin in VMD 1.9.4^65^. Subsequently, extra water molecules were deleted, and the water boxes were shaped to fit in the periodic hexamer lattice of dimensions: *a* = *b* = 277 (except E45A^a^: 263) and *c* = 235 (except E45A^b^: 252) Å, with unit cell angles *α* = *β* = 90° and *γ* = 120°. The solvated systems were then neutralized by adding sodium and chloride ions and the total concentration of NaCl was set to 150 mM using Autoionize plugin (version 1.3) in VMD. The resulting models contained ~1.6 million atoms, including protein, water, and ions.

#### Molecular dynamics simulations

Molecular dynamics (MD) simulations for each of the models were performed using NAMD 2.12^66^ and CHARMM36 force field^67^. Each model was initially subjected to an energy minimization and followed by a thermalization of 10,000 steps, while applying harmonic restrains of 10 kcal·mol^−1^·Å^−2^ on the protein heavy atoms. After heating, the whole system was subjected to three steps of equilibration of 0.5 ns where the harmonic restraints were gradually released. Production simulations were carried out using a Langevin thermostat at 310 K and a Langevin piston barostat at 1.0 atmosphere; a v-RESPA integrator was employed with an internal time step of 2 fs and electrostatic interactions were treated using the PME (particle-mesh Ewald) algorithm with a 1.2 nm cutoff. Long-range interactions were updated every 4 fs while non-bonded interactions were recalculated every 2 fs. The SHAKE algorithm was applied to all hydrogen bonds. Simulations were performed with a total of at least 130 ns for each model in the Blue Waters super-computer.

#### Molecular dynamics simulations—Tempering

Tempering simulations of WT and mutant E45A^a^, E45A^b^ and E45A/R132T hexameric lattices were accomplished using NAMD2.14. Utilizing the equilibrated systems, prepared according to above section, a series of constant-pressure constant-temperature (NPT) simulations were performed at varying temperatures; for all CA lattices, temperatures ranging from 310 K to 500 K, with a stride of 10 K, were employed. Target temperatures for the Langevin thermostat and Langevin piston barostat were set to the relevant temperature for each simulation. Tempering simulations utilized the CHARMM36m protein force field. Target pressures for the barostat were maintained at 1.0 atmosphere for all simulations. A v-RESPA integrator was employed with an internal timestep of 2 fs, and long-range electrostatics were computed using the particle mesh Ewald (PME) algorithm, the latter utilizing a distance cutoff of 1.2 nm. Long-range interactions were computed every 4 fs, while short-range interactions were computed every 2 fs. The SHAKE algorithm was employed to constrain all bonds to hydrogen.

#### Analysis of lattice thermal stability

To establish the thermal stability of the WT and CA mutant lattices, each dimer interface in the 2 × 3 × 3 hexameric systems was identified and aligned to its reference dimer modeled at a temperature of 310 K. Following alignment, the RMSD (measured in Å) among backbone heavy atoms in the C-terminal domains of monomers comprising the dimer interface was measured. A dimer interface was considered melted, or disrupted, when the RMSD was greater than or equal to 3.5 Å from its reference dimer. The fraction of melted dimers, taken as the number melted divided by the total number of dimer interfaces in the system, was then computed for each CA lattice construct at every temperature simulated. The T_m_ was then determined as the temperature where the fraction melted was equal to 0.5; that is, the temperature where half of the dimer interfaces deviated from their reference interface by more than 3.5 Å.

#### Analysis of ion and water transport through CA hexamers

Analysis of MD trajectories were performed using VMD^65^ and NAMD^66^. We began the analysis of the trajectories by calculating the exchange rates of ions and water molecules. First, based on the definition of the exterior and interior regions of the capsid, water molecules and ions in the exterior/interior region were labeled as exterior/interior molecules. Subsequently, the number of exterior/interior molecules at a reference time t_0_, which were found at a later time t > t_0_ on the opposite region of the capsid, interior/exterior, were counted. Importantly, the slopes of the counts versus time constitute the exchange rates for ion and water molecules (in number of molecules per ns) and were calculated.

#### Flexibility of WT and mutant CA hexamers

Flexibility of the capsid protein was assessed by means of the root mean squared fluctuation (RMSF) calculated using VMD^65^. Rotations and translations of the capsid lattices during the simulation were removed by aligning all the monomers to reference structures. The resulting RMSFs of Cα were averaged over the eighteen monomers present in each simulation.

#### Interaction energies between and within CA hexamers

The pair interaction function implemented in NAMD^66^ was used to calculate the electrostatic and Van der Waals interaction energies. Inter-hexamer interaction energies were calculated between a central hexamer and six surrounding hexamers and averaged during the course of the simulation.

#### Electrostatics of CA hexamer

Electrostatic calculations of the hexamer models were performed using the APBS package^68^. Starting from a 3 × 3 lattice of hexamers, the electrostatic density maps of each model were evaluated using an asynchronous parallel calculation. The electrostatic surfaces of the hexamer models were then visualized using VMD.

### Reporting summary

Further information on research design is available in the Nature Portfolio Reporting Summary linked to this article.

### Supplementary information

Supplementary Information

Reporting Summary

### Source data

Source Data

## Data Availability

The authors declare that the data supporting the findings of this study are available within the paper and its Supplementary Information file and from the corresponding author upon request. X-ray crystal structure coordinates and structure factor data have been deposited into the RCSB Protein Data Bank (PDB) under accession codes 6AY9 (WT CA/CPSF6), 6AYA (WT CA/Nup153), 6B2G (P38A CA), 6B2H (P38A/T216I CA), 6B2I (E45A^a^ CA), 6B2J (E45A^b^ CA), 6B2K (E45A/R132T CA). The manuscript also refers to existing crystal structures 4XFX (WT CA) and 4XFZ (WT CA/PF74). The molecular dynamics datasets generated and analyzed during the current study are available in the Zenodo repository at 10.5281/zenodo.8180089. The source data underlying Fig. 4 and Supplementary Fig. 8c, 9b, c, 13a–c, 14a–c, and 18 are provided as a Source data file.
