# Supplementary material for: Multidisciplinary studies with mutated HIV-1 capsid proteins reveal structural mechanisms of lattice stabilization
Source: Nat Commun. 2023 Sep 12;14:5614. doi: 10.1038/s41467-023-41197-7 (PMC10497533; doi:10.1038/s41467-023-41197-7)
Supplement: Supplementary file 1 — Supplementary Information [file 41467_2023_41197_MOESM1_ESM.pdf]

Multidisciplinary studies with mutated HIV-1 capsid proteins reveal structural mechanisms of lattice stabilization

Anna T. Gres, Karen A. Kirby, William M. McFadden, Haijuan Du, Dandan Liu, Chaoyi Xu, Alexander J. Bryer, Juan R. Perilla, Jiong Shi, Christopher Aiken, Xiaofeng Fu, Peijun Zhang, Ashwanth C. Francis, Gregory B. Melikyan, and Stefan G. Sarafianos*

Supplementary Information

1. Supplementary Figures and Legends
2. Supplementary Tables
3. Uncropped gel from Supplementary Figure 8c
4. Supplementary References

1. Supplementary Figures and Legends

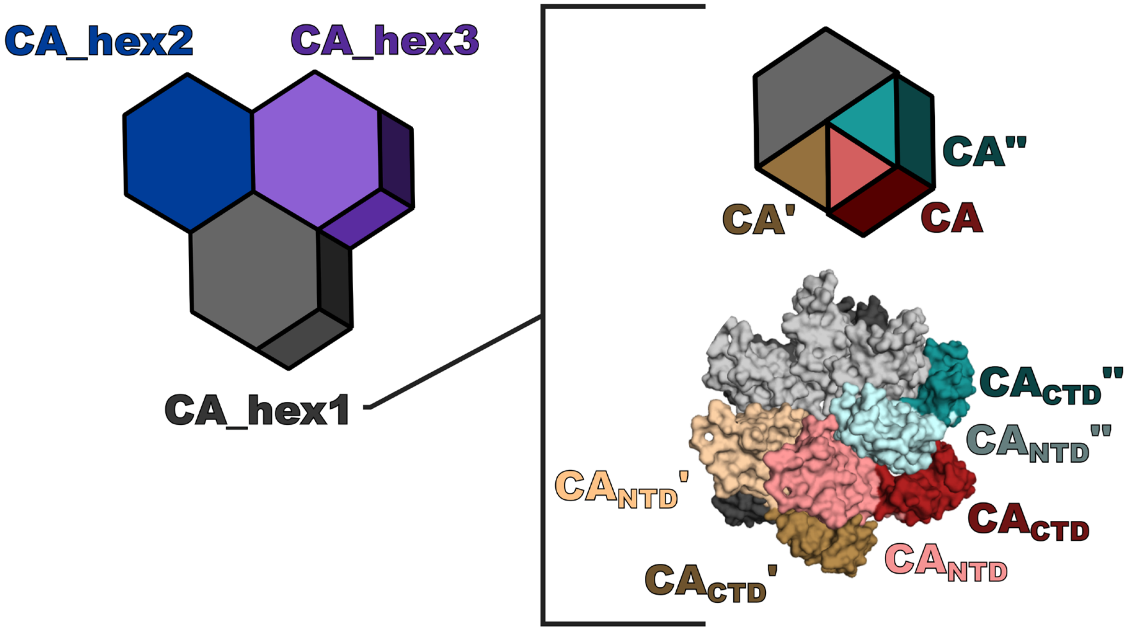

Supplementary Figure 1. Cartoon schematic of mature HIV-1 CA and related nomenclature.

Full hexagons represent mature CA hexamers assembled by six CA monomers. Left represents hexamer-hexamer interactions at the 3-fold interface, labeled as CA_hex1, CA_hex2, and CA_hex3 and colored as grey, navy, and purple, respectively. Top right represents a single hexamer (CA_hex1) with three adjacent CA monomers, CA”, CA, CA’, represented as triangles and colored in cyan, red, and brown, respectively. Bottom right shows a model of the wild-type CA hexamer structure (PDB ID: 4XFX) with colors corresponding to the above cartoon. N-terminal domains (CA_{NTD}s) are shown in light hues, C-terminal domains (CA_{CTD}s) are shown in dark hues.

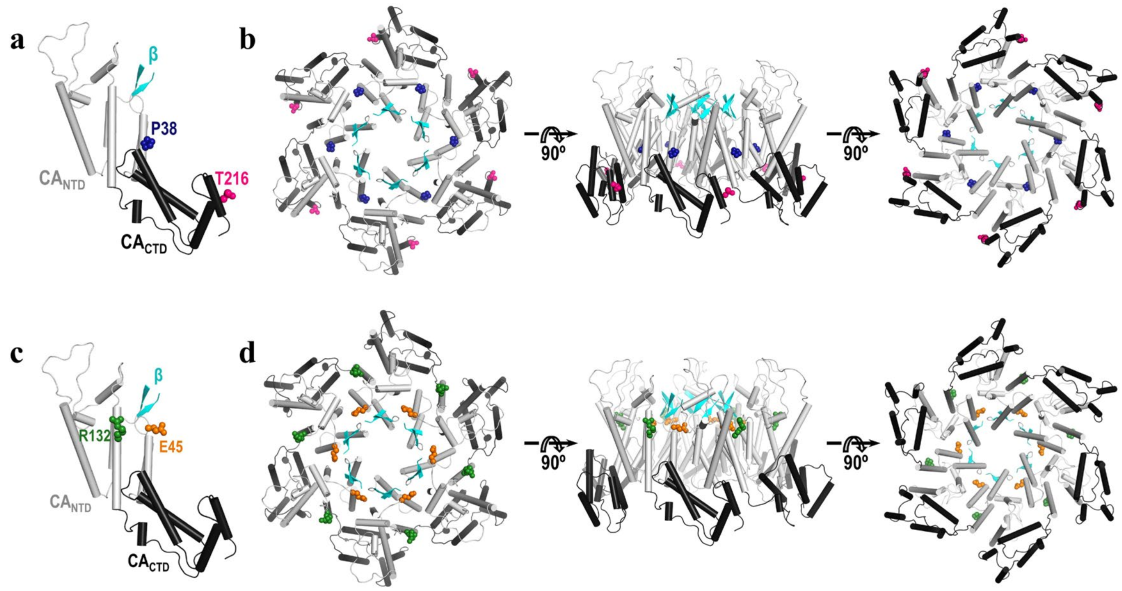

Supplementary Figure 2. Mutation sites in the wild-type full-length HIV-1 capsid protein (CA WT).

(**a**) Locations of mutation sites P38 (dark blue spheres) and T216 (pink spheres) in CA WT monomer and (**b**) hexamer shown with alternate orthogonal views. (**c**) Locations of mutation sites E45 (orange spheres) and R132 (green spheres) in CA WT monomer and (**d**) hexamer. CA_{NTD}s are in light grey, CA_{CTD}s in dark grey, N-terminal β-hairpin in light blue.

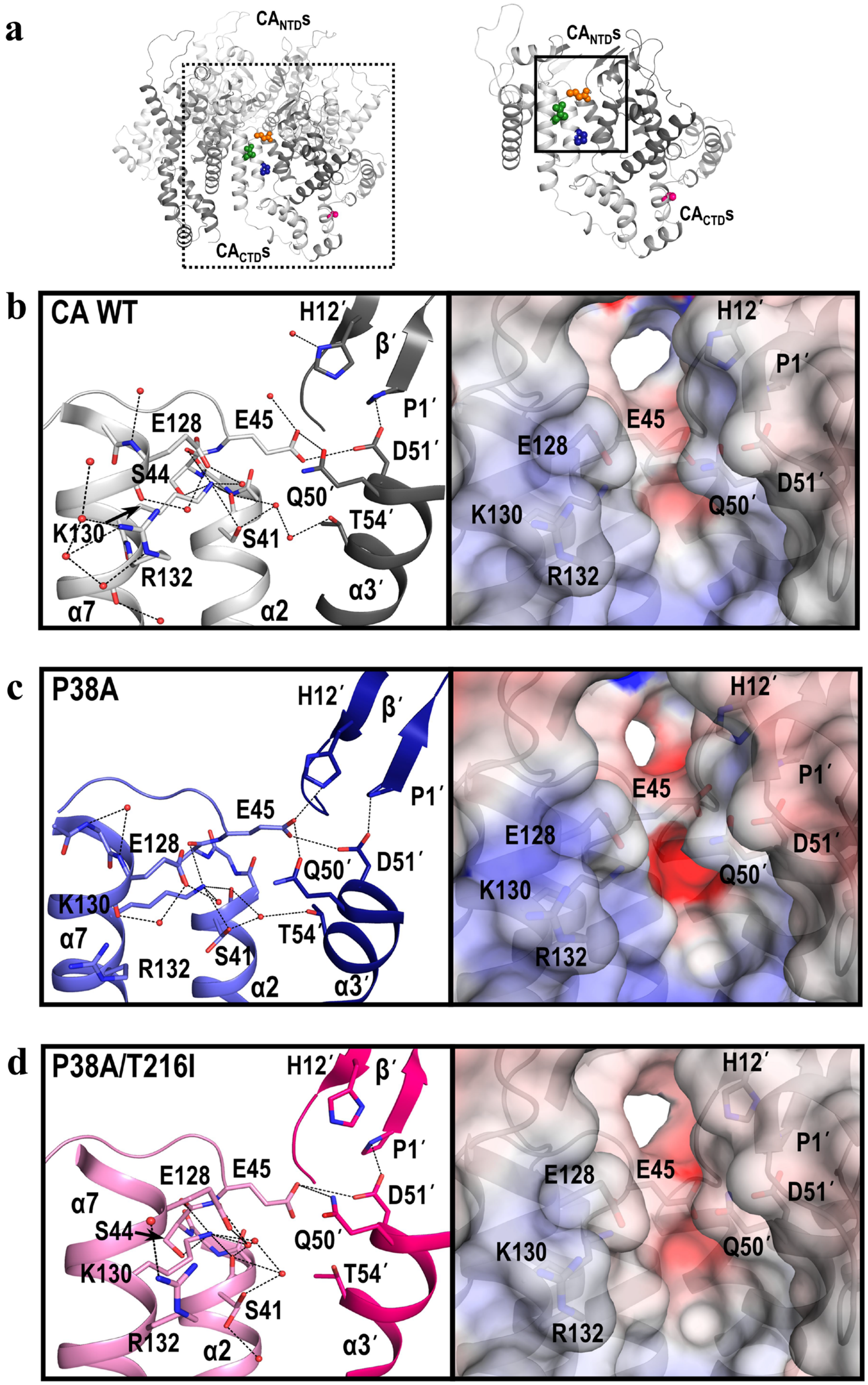

Figure continued on next page

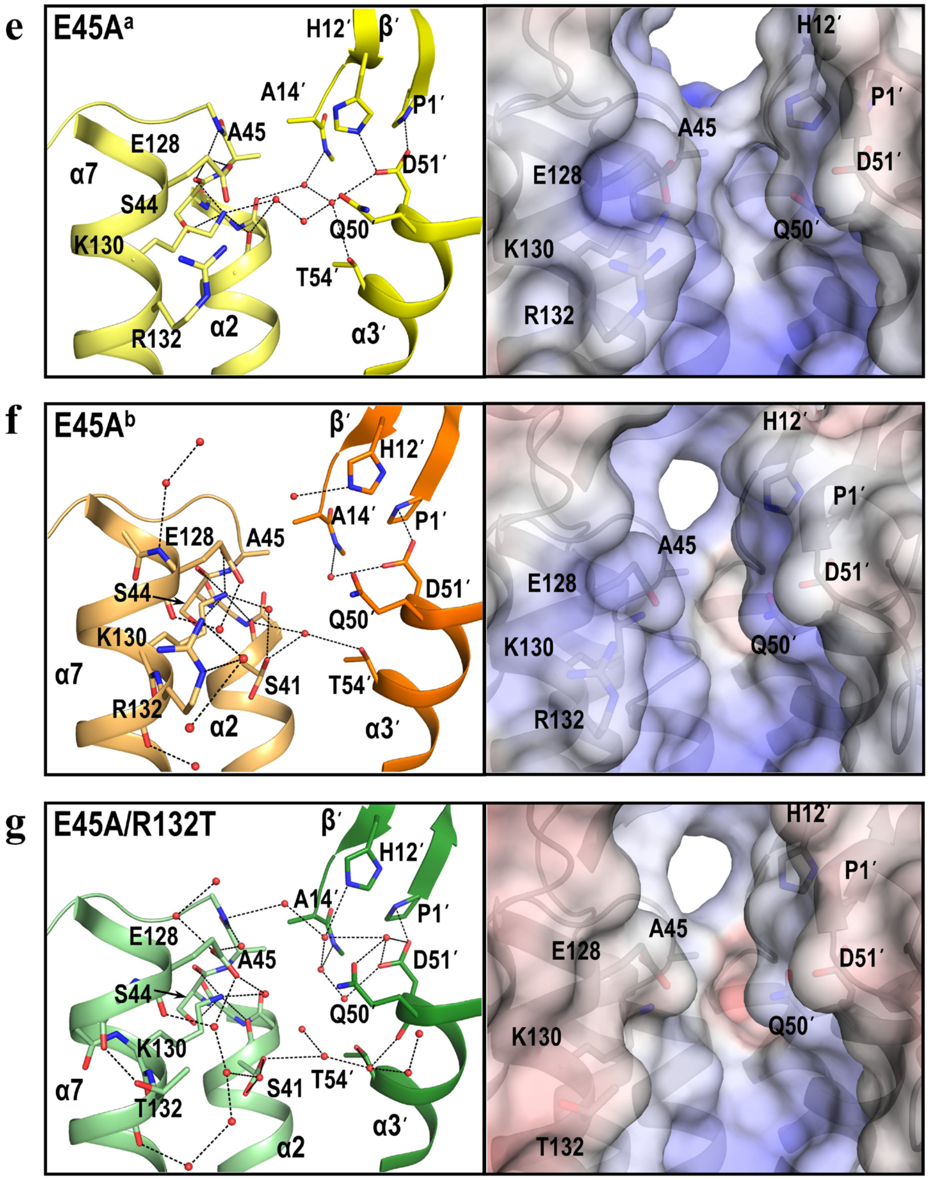

Supplementary Figure 3. The effects of mutations on polar and water-mediated contacts around residue 45 and the salt bridge between P1 and D51.

(**a**) Orientation of CA WT hexamer with the side view of one representative monomer (white) and its interaction with the adjacent subunit (grey) outlined in dashed line and enlarged. Locations of mutation sites P38 (blue), T216 (pink), E45 (orange), and R132 (green) are shown as spheres. The effects of mutations on the region around residue 45 and the salt bridge between P1 and D51 (enlarged views of the boxed region, solid line) are shown in (**b-g**). Polar and water-mediated contacts in CA WT (**b**), P38A (**c**), P38A/T216I (**d**), E45A[a] (**e**), E45A[b] (**f**), and E45A/R132T (**g**). Black dashed lines indicate atoms within 3.6 Å. Waters are shown as red spheres. Selected side chains are shown explicitly and labeled. Surface representation of respective views are colored according to electrostatic potential from −10 k_BT/e (red) to +10 k_BT/e (blue).

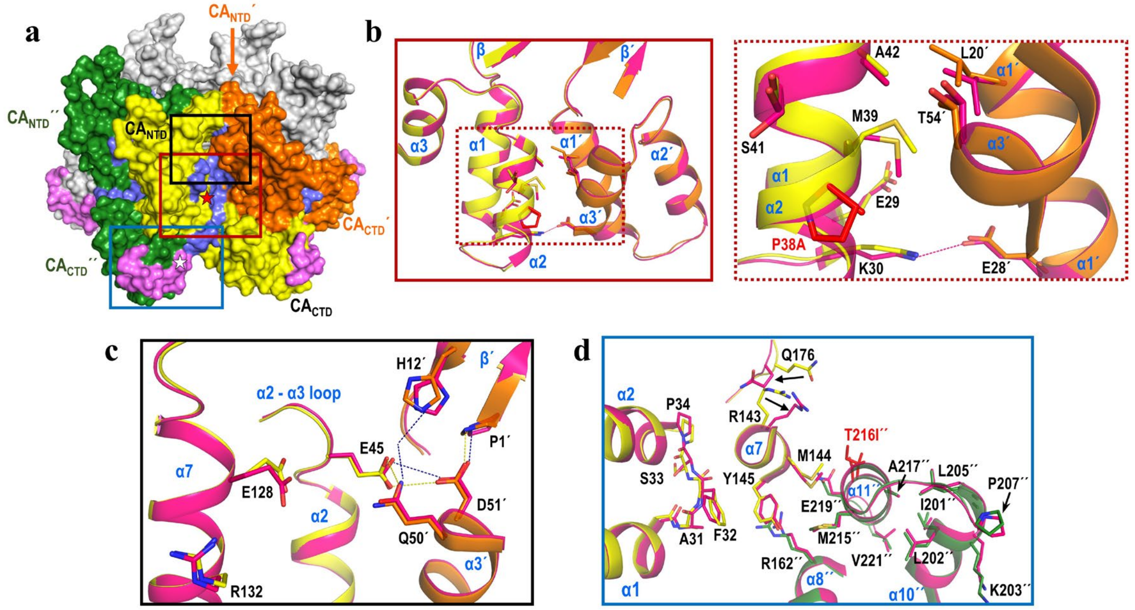

Supplementary Figure 4. Structural changes associated with P38A/T216I mutations.

(**a**) A CA hexamer is shown in surface view representation with three neighboring intra-hexamer CA monomers colored in orange (subunit ´), yellow (subunit without prime symbol), and green (subunit ´´); the other three are shown in gray. Select mutation sites in neighboring subunits are marked with red (A38) and white (I216´´) stars. Regions likely affected by the P38A mutation are shown in light blue surface; residues likely affected by the T216I´´ mutation are shown in magenta surface. The regions in red, black, and blue boxes are depicted in (**b**), (**c**), and (**d**), respectively. (**b-d**) Superposition of WT (cartoon ribbons of three neighboring subunits colored in green, yellow, and orange) and P38A/T216I (in pink) CA. Mutations alter CA$_{NTD}$-CA$_{NTD}$ (**b, c**) and CA$_{NTD}$-CA$_{CTD}$ interfaces (**d**). Specific residues affected by P38A and T216I mutations (in red) are shown as sticks. Dashed lines are shown between residues that are within 4 Å. For clarity, residues G220´´, A204´´ are not shown. Box colors in (**b-d**) correspond to the boxed regions in panel (**a**). Dashed box in (**b**) is an insert of a region within the other box in (**b**).

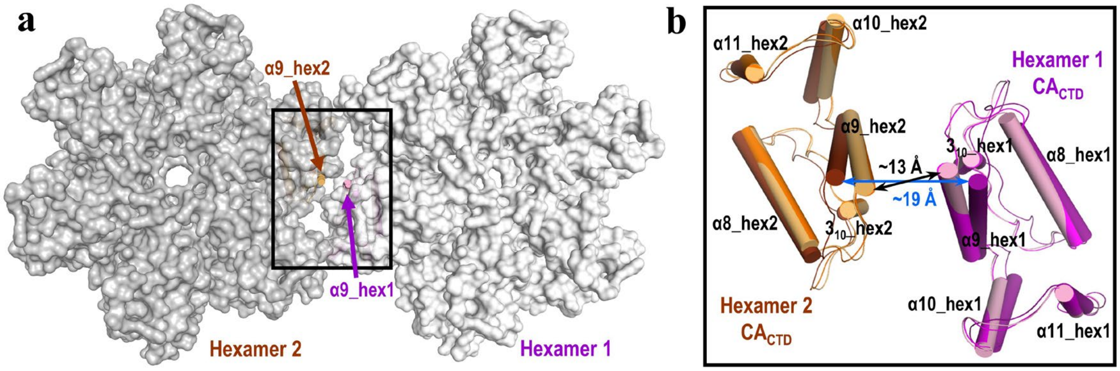

Supplementary Figure 5. E45A can cause rearrangements at inter-hexamer interfaces, while addition of the R132T mutation reverses these changes in E45A/R132T.

(**a**) Two neighboring hexamers of E45A[a] CA are shown in surface view. Least squares superposition (alignment based on residues 17–143) of E45A[a] (dark brown and dark purple CA_{CTD}s) with WT (light orange and light pink CA_{CTD}s) and E45A/R132T (orange and magenta CA_{CTD}s) structures. (**b**) Enlarged view of the boxed region in (**a**) shows changes in the position of helices α9, 3_{10}, α10, and α11 in both hexamers (marked as hex1 and hex2). The blue arrow indicates the distance between α9_hex1 and α9_hex2 in E45A[a], while the black arrow indicates the distance between α9_hex1 and α9_hex2 in WT and E45A/R132T. Addition of the R132T mutation reverses the effect of the E45A mutation at these interfaces.

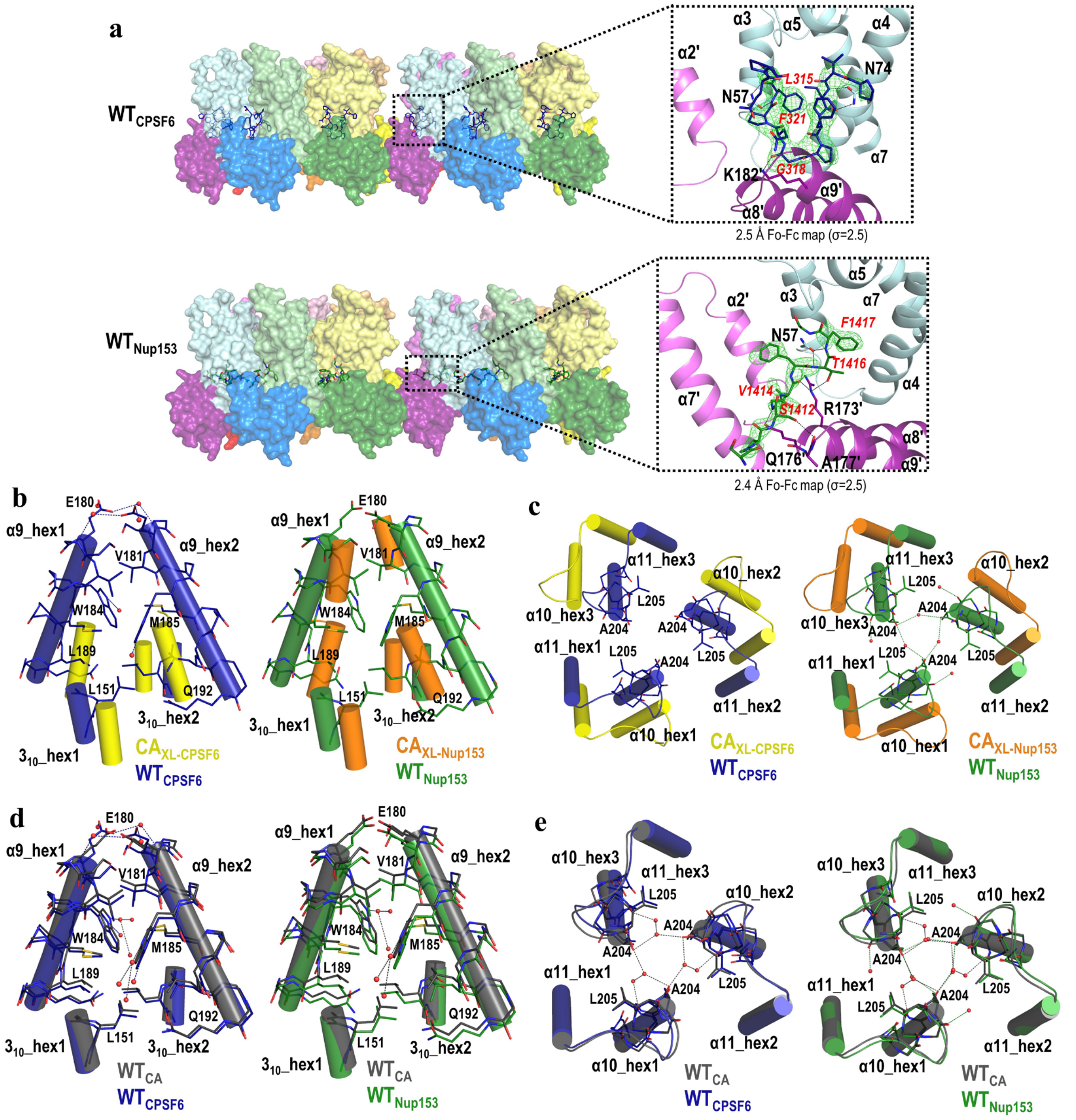

Supplementary Figure 6. Crystal structures of native WT$_{CPSF6}$ and WT$_{Nup153}$. (a) CPSF6 and Nup153 peptides bind at the PF74 binding pocket, which is between the CA$_{NTD}$ of one CA monomer (no prime) and the CA$_{CTD}$ of a neighboring CA monomer within a hexamer (denoted by prime symbols). Enlarged views show the details of how CPSF6 (blue sticks) and Nup153 (green sticks) bind at the PF74 binding pocket. Fo-Fc maps at σ=2.5 are shown in green. Peptide labels are italicized and colored in red. (**b**) Comparison of native WT$_{CPSF6}$ (blue) *vs.* cross-linked CA in complex with CPSF6 (CA$_{XL-CPSF6}$, yellow, left panel) and native WT$_{Nup153}$ (green) *vs.* cross-linked CA in complex with Nup153 (CA$_{XL-Nup153}$, orange, right panel) demonstrates significant changes at the 2-fold inter-hexamer interface. (**c**) Comparison of native WT$_{CPSF6}$ (blue) *vs.* cross-linked CA in

complex with CPSF6 ($CA_{XL\text{-}CPSF6}$, yellow, left panel) and WT_{Nup153} (green) *vs.* cross-linked CA in complex with Nup153 ($CA_{XL\text{-}Nup153}$, orange, right panel) demonstrates significant changes at the 3-fold inter-hexamer interface. (**d**) Comparison of native WT CA (WT_{CA}, gray) *vs.* native WT_{CPSF6} (blue, left panel) and native WT_{CA} (gray) *vs.* native WT_{Nup153} (green, right panel) reveal subtle changes at the 2-fold inter-hexamer interface. (**E**) Comparison of native WT_{CA} (gray) *vs.* native WT_{CPSF6} (blue, left panel) and native WT_{CA} (gray) *vs.* native WT_{Nup153} (green, right panel) reveal subtle changes at the 3-fold inter-hexamer interface.

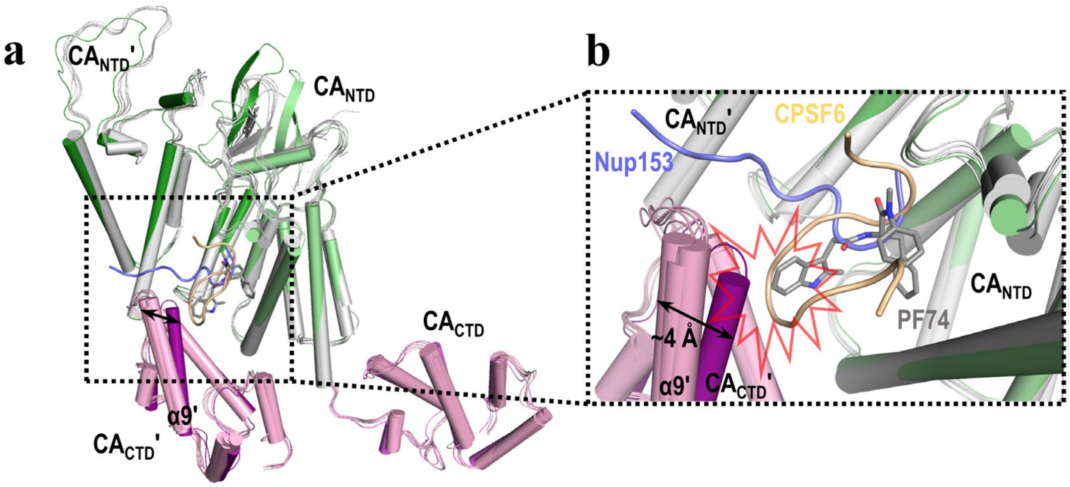

Supplementary Figure 7. Conformational changes caused by E45A affect access to the PF74/CPSF6/Nup153 binding pocket. (**a**) Least squares superposition (alignment based on residues 17–145) of E45A[a] (green CA_{NTD}s, purple CA_{CTD}s) with WT CA (CA), E45A/E132T CA, WT CA in complex with a CPSF6 peptide (WT_{CPSF6}), WT CA in complex with a Nup153 peptide (WT_{Nup153}), WT CA in complex with PF74 (WT_{PF74}), (gray CA_{NTD}s, light pink CA_{CTD}s). Two intra-hexamer CA monomers are shown (neighboring subunit is marked by a prime symbol). (**b**) Enlarged view of the boxed region in (**a**) showing the entrance to the PF74/CPSF6/Nup153 binding pocket. The change in position of helix α9 in the neighboring subunit (marked with a prime symbol) between E45A[a] (dark purple CA_{CTD}) and the other structures (light pink CA_{CTD}s) is noted with a black arrow. The red explosion graphic denotes regions of steric clash between the CPSF6 peptide and PF74 with α9' of E45A[a] CA.

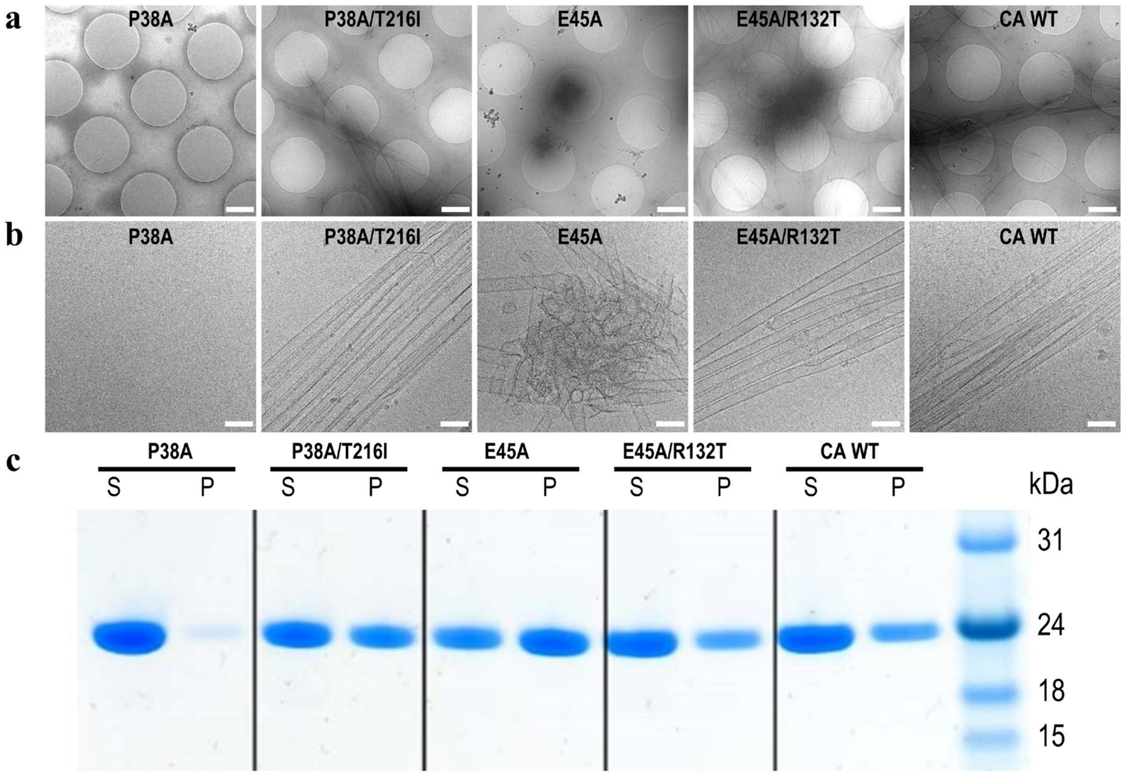

Supplementary Figure 8. Effects of capsid mutations on assembly.

(**a-b**) Cryo-EM analysis of CA mutant assemblies. Projection images were recorded at low (**a**) and high (**b**) magnifications from the corresponding samples as indicated. Scale bars, 1 μm in (**a**), and 100 nm in (**b**), respectively. (**c**) Pelleting assay for CA mutant assemblies. Four CA mutants and CA WT are labeled. 'S' and 'P' stand for the supernatant and pellet from each sample. Protein products are visualized by Coomassie Blue staining. Molecular weight markers are labeled on the right. Experiments were performed as three biological replicates, with representative experiments shown above. An image of the uncropped gel in (**c**) is shown in the Source Data file and below on page 42.

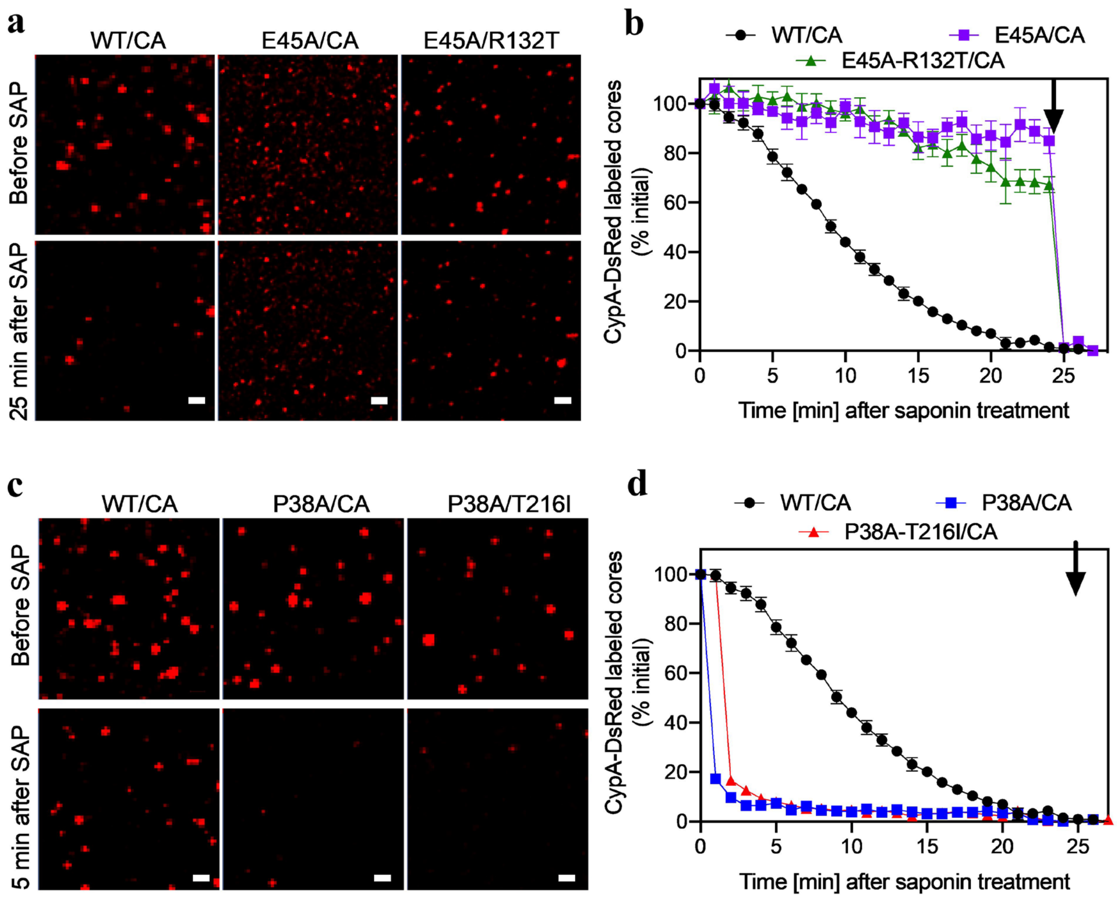

Supplementary Figure 9. Effects of capsid mutations on HIV-1 core stability.
An *in vitro* HIV-1 core stability assay was performed using INsfGFP (green) and CypA-DsRed (red) labeled pseudoviruses immobilized on poly-L-lysine coated coverslips and permeabilized by brief exposure to saponin (see Methods). (**a, c**) Images showing CypA-DsRed puncta immediately before (top panel) and 25 min or 5 min after (bottom panel) virus membrane permeabilization with saponin (SAP). (**b, d**) The kinetics of CypA-DsRed loss from INsfGFP-labeled HIV-1 cores over time at room temperature. Arrows in (**b**) and (**d**) mark the time of CsA (5 μM) addition at 25 min post-permeabilization to displace CypA-DsRed from remaining HIV-1 cores. Plots are means and standard errors from 4 independent experiments; for each experiment, 4 fields of view were analyzed. Scale bar in (**a, c**) is 2 μm.

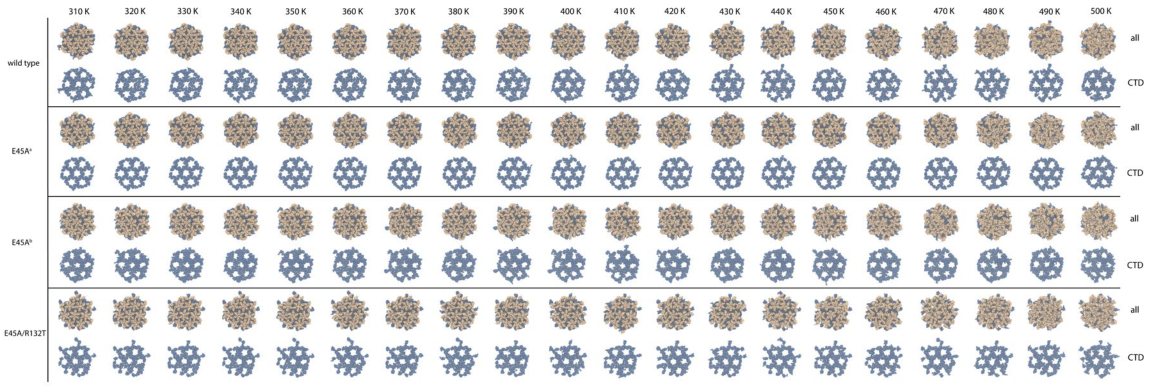

Supplementary Figure 10. ***In silico*** **thermal stability assay of WT and mutant CA lattices.**

Snapshots of both NTDs and CTDs for WT, E45Aa, E45Ab, and E45A/R132T lattices across every simulated temperature in the *in silico* thermal stability assay.

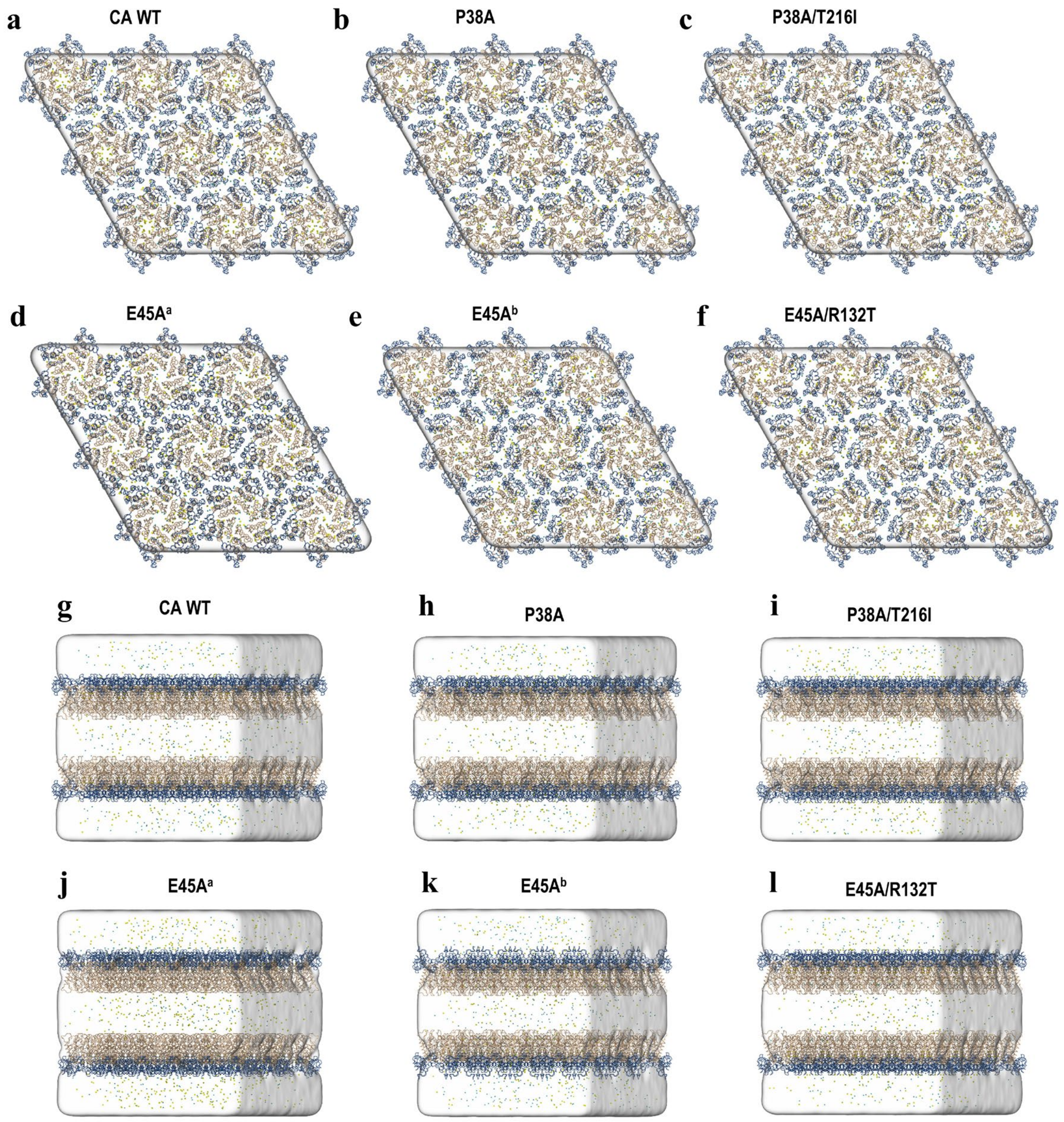

Supplementary Figure 11. Hexamer models of CA WT and mutant proteins.

(**a**-f) Top views and (**g-l**) side views of CA WT, P38A, P38A/T216I, E45A^{a}, E45A^{b}, and E45A/R132T hexamers, respectively. The CA_{NTD}s are in light brown and CA_{CTD}s in blue.

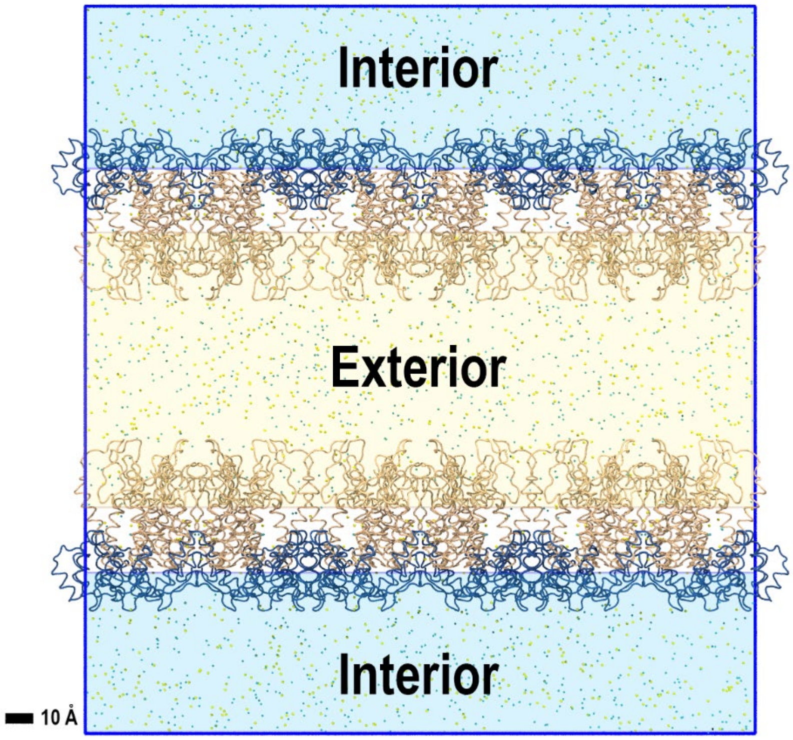

Supplementary Figure 12. Exterior and interior regions of CA.

Sodium and chloride ions are represented by yellow and cyan dots, respectively. The CA_{NTD}s are in light brown and CA_{CTD}s in blue.

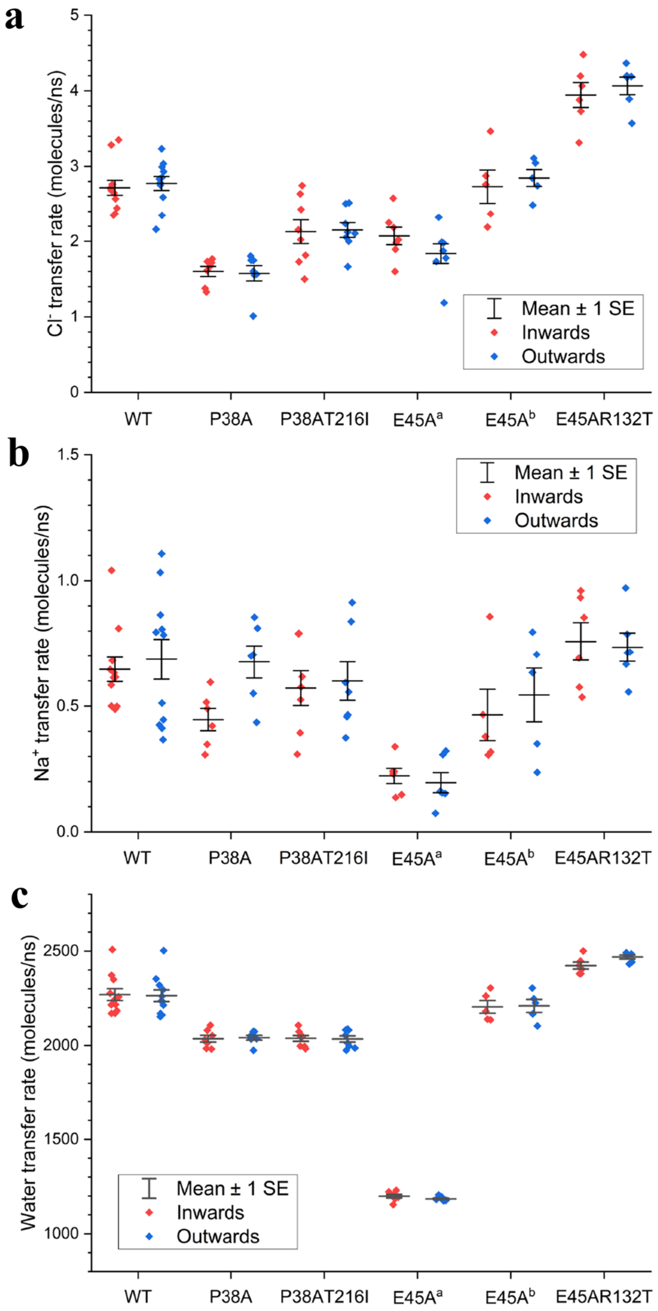

Supplementary Figure 13. Ions and water transfer rates of CA hexamers.

(**a**) Chloride ion transfer rates of hexamers. (**b**) Sodium ion transfer rates of hexamers. (**c**) Water transfer rates of hexamers. For all panels and for each construct denoted on the x-axis, rates were computed from intervals of 1,000 frames, yielding n=12 inward and outward rate measurements per simulation, over which means and standard errors were computed.

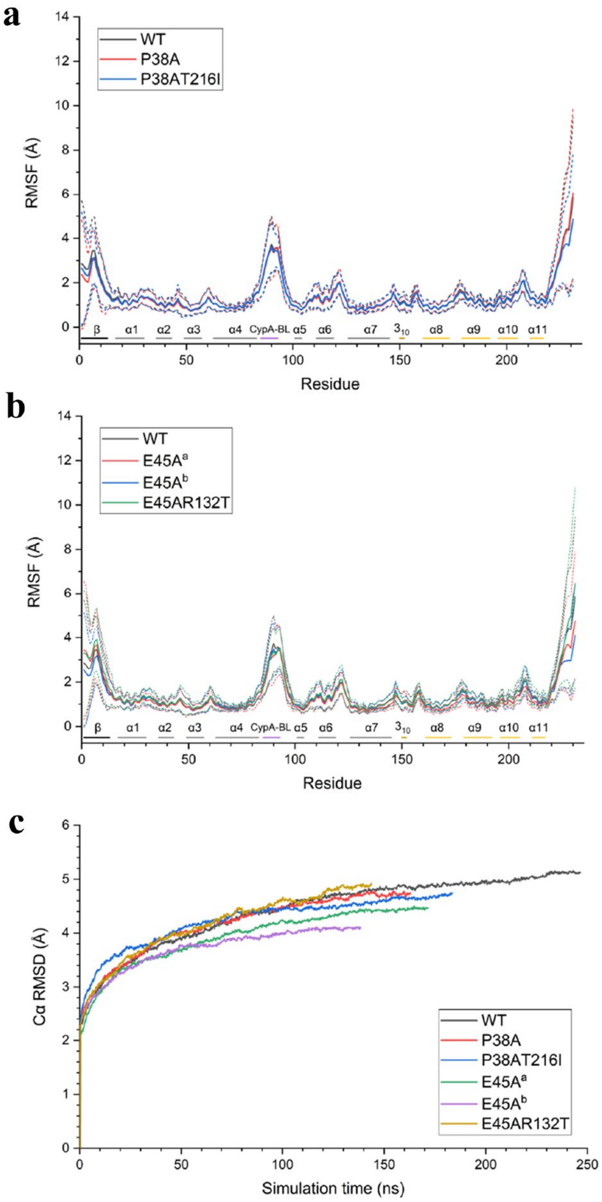

Supplementary Figure 14. C-alpha RMSF of CA WT and mutants.

(**a**) RMSF of CA WT, E45A[a], E45A[b], and E45A/R132T mutants. (**b**) RMSF of CA WT, P38A, and P38A/T216I mutants. Short dash lines represent ± standard deviation. The lines below represent the sequence positions of key secondary structure elements (helices, loops) in CA WT. Helices in CA_{NTD}: helix α1 (residues 17 to 30), helix α2 (36 to 43), helix α3 (49 to 57), helix α4 (63 to 83), helix α5 (101 to 104), helix α6 (111 to 119) and helix α7 (126 to 145), are in gray; helices in CA_{CTD}: helix α8 (161 to 173), helix α9 (179 to 192), helix α10 (196 to 205) and helix α11 (211 to 217), are in orange; β-hairpin (1 to 13) is in black, the purple line stands for CypA-binding loop (residues 85 to 93) and the 3_{10} helix (150 to 152) is in orange. (**c**) Ca RMSD of CA hexamers.

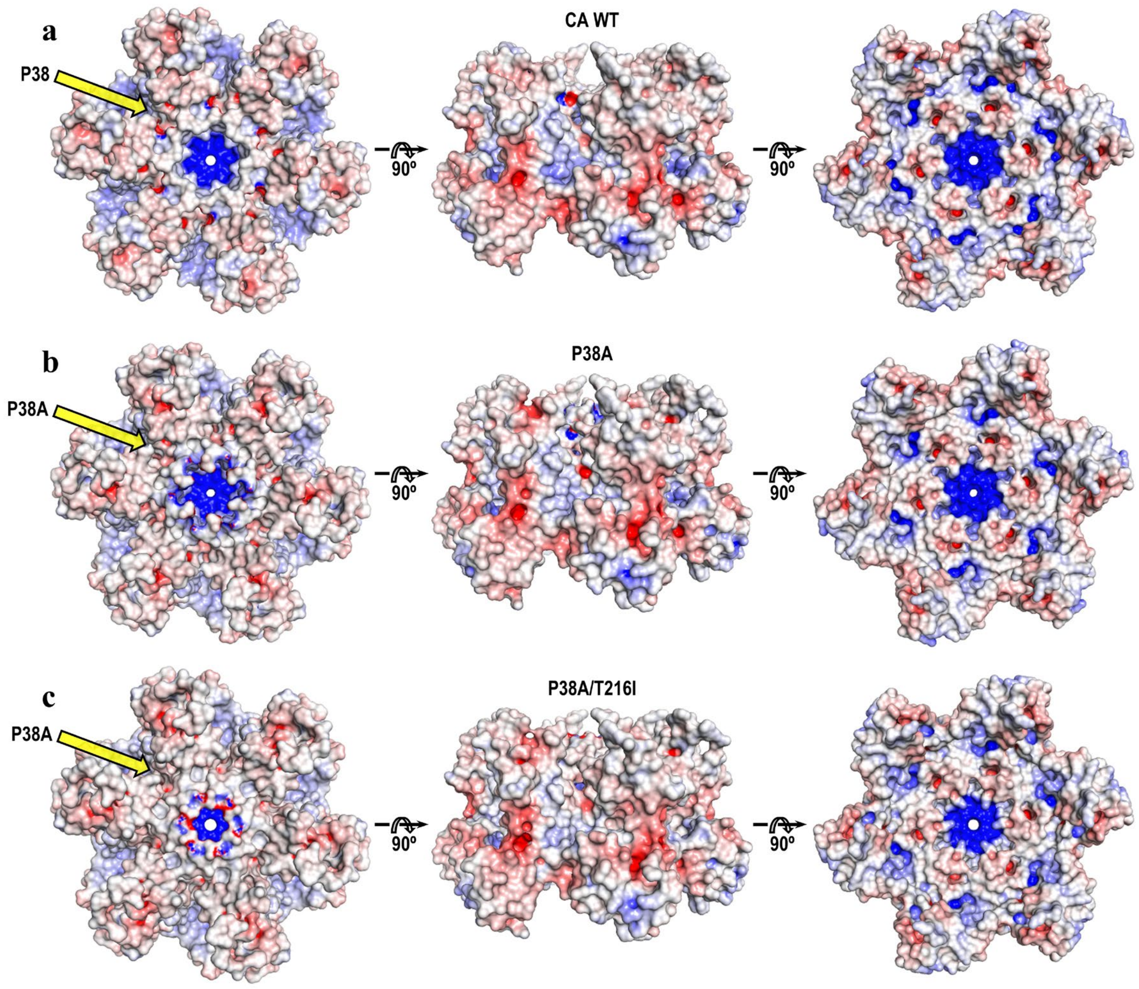

Supplementary Figure 15. Surface electrostatic potential of CA WT, P38A, and P38A/T216I.

Surface representation of CA WT (**a**), P38A (**b**), and P38A/T216I (**c**) hexamers with alternate orthogonal views colored according to electrostatic potential from −10 k_BT/e (red) to +10 k_BT/e (blue). The position of P38 or P38A is shown by the yellow arrow.

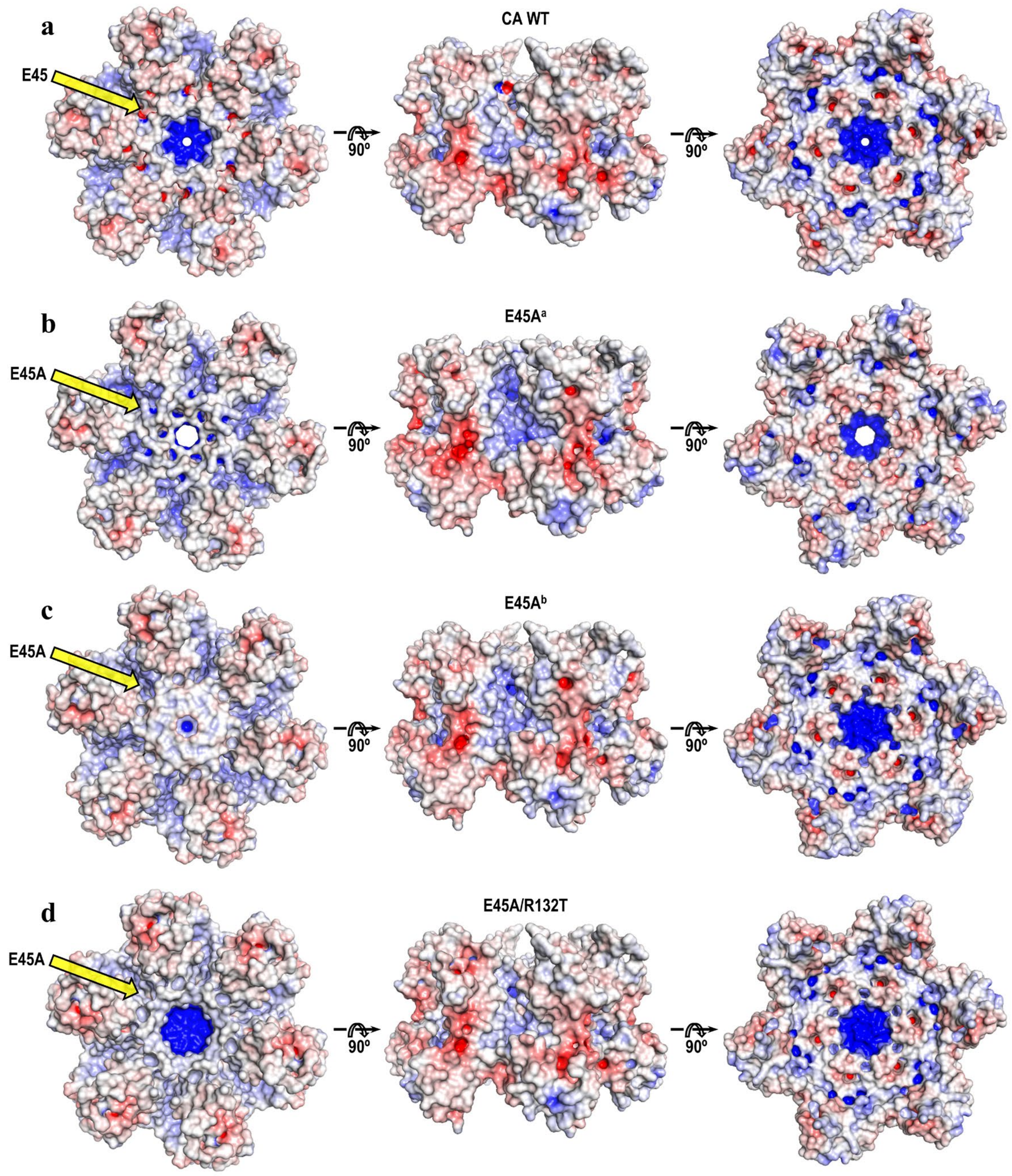

Supplementary Figure 16. Surface electrostatic potential of CA WT, E45A, and E45A/R132T.

Surface representation of CA WT (**a**), E45A[a] (**b**), E45A[b] (**c**), and E45A/R132T (**d**) hexamers with alternate orthogonal views colored according to electrostatic potential from −10 k_BT/e (red) to +10 k_BT/e (blue). The position of E45 or E45A is shown by the yellow arrow.

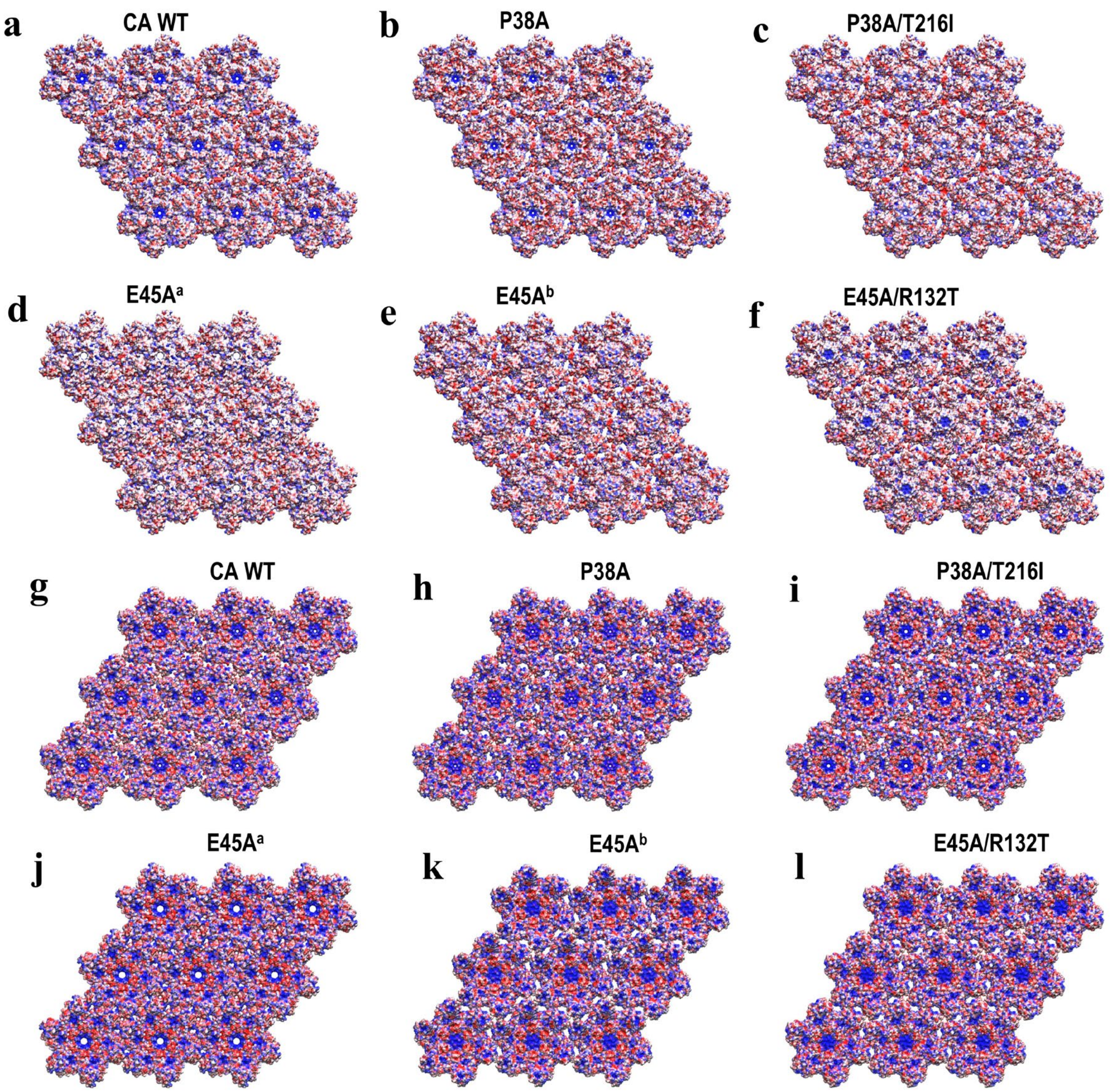

Supplementary Figure 17. Electrostatic properties of hexamers.

(**a-f**) top views and (**g-l**) bottom views of CA WT, P38A, P38A/T216I, E45A[a], E45A[b], and E45A/R132T hexamers, respectively. The electrostatic potential was colored from red (−10 k_BT/e) to blue (+10 k_BT/e).

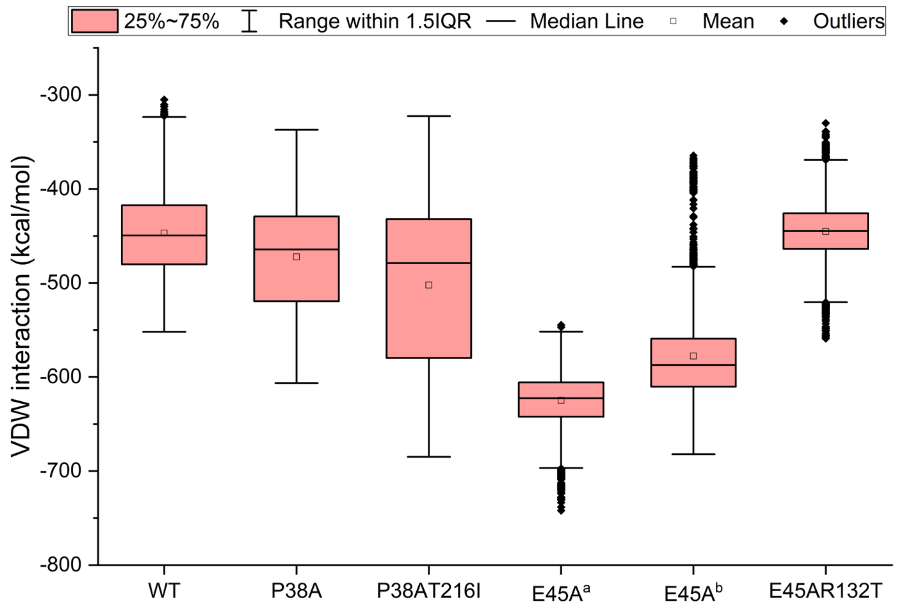

Supplementary Figure 18. Van der Waals inter-hexameric interaction energies.

Distributions of Van der Waals interaction energies of inter-hexamer interactions. The analysis considered the interaction energies of sets of atoms, situated at inter-hexameric interfaces and identified via distance cutoff, over which the statistics shown above were computed. For wild type, n=6,151 atoms; for P38A, n=4,074 atoms; for P38AT16I, n=4,585 atoms; for E45A[a], n=3,362 atoms; for E45A[b], n=4,287 atoms; for E45AR132T, n=3,597 atoms.

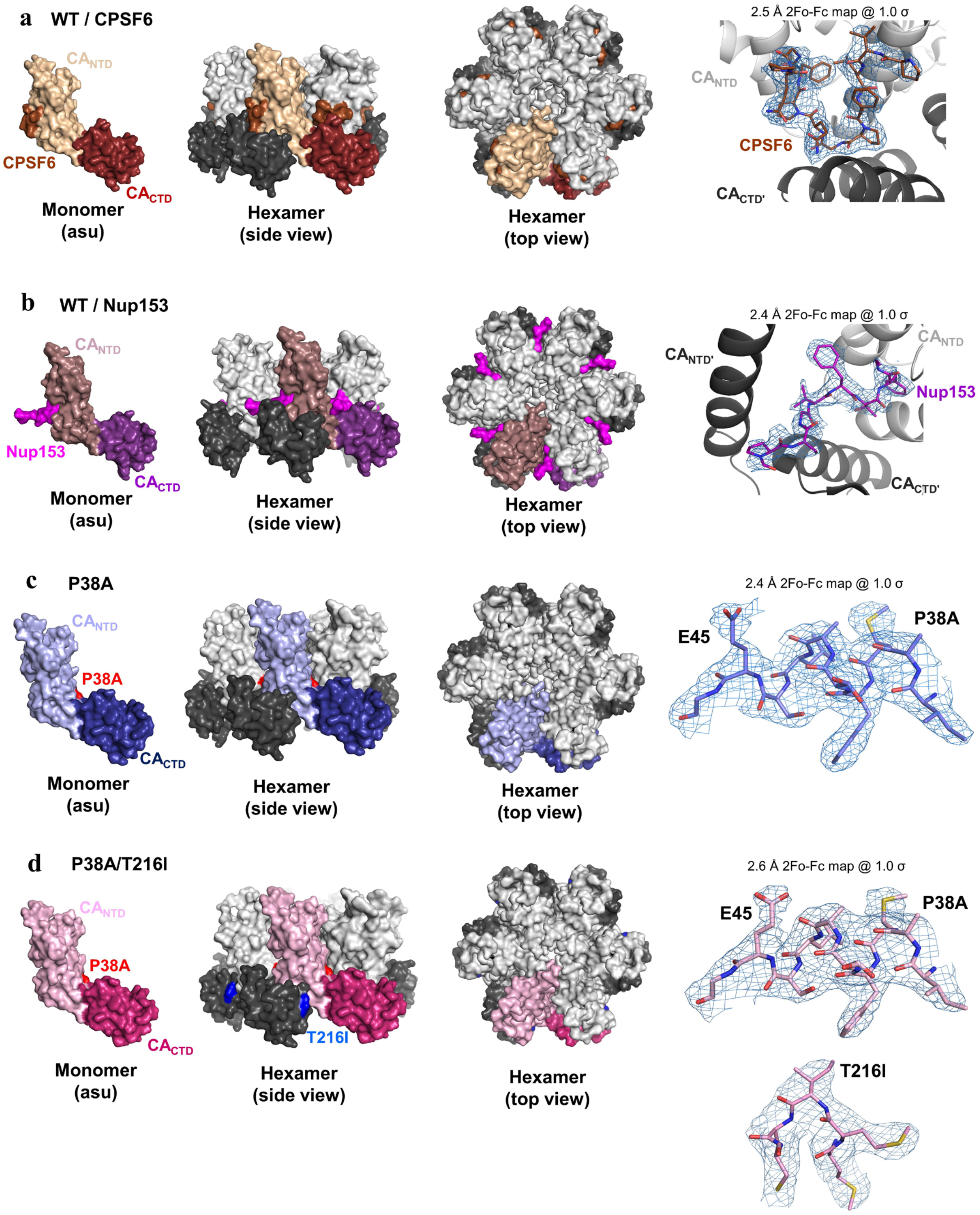

Figure continued on next page

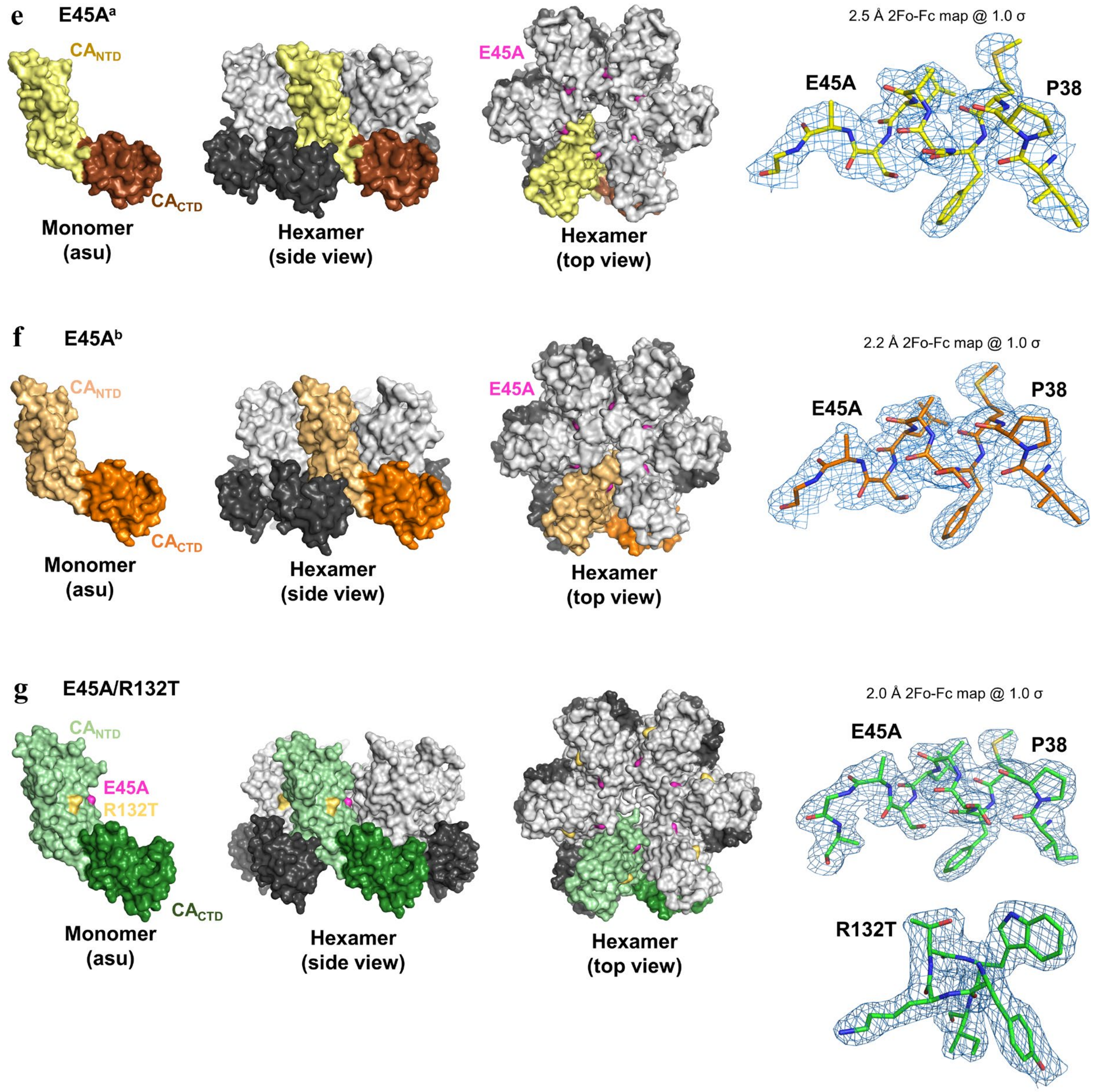

Supplementary Figure 19. Crystal structures of CA mutants and WT / CPSF6 or Nup153 peptides. X-ray crystal structures of WT CA / CPSF6 peptide (**a**), WT CA / Nup153 peptide (**b**), and P38A (**c**), P38A/T216I (**d**), E45A[a] (**e**), E45A[b] (**f**), E45A/R132T (**g**) CAs. Left panel shows the asymmetric unit (asu), a CA monomer, which assembles into the hexameric biological assembly (middle two panels, side and top views). Structures in the first three panels are represented in surface view. The right panel shows electron density (2Fo-Fc maps shown in blue mesh contoured at σ=1.0) around the bound peptides in (**a-b**) or mutations of interest in (**c-g**). Colors as noted in figure; light gray surface represents symmetry-related N-terminal domains (CA_{NTD}) in the biological assembly, while dark gray surface represents the symmetry-related C-terminal domains (CA_{CTD}) in the biological assembly. Some mutations are obstructed from view in the specific orientations above.

2. Supplementary Tables

Supplementary Table 1. Available biological data for CA WT and mutants.

Assay	WT_{CA}	P38A	P38A/T216I	E45A	E45A/R132T	Interpretations, references
In vitro						
**Isolation of HIV-1 cores by centrifugation via a detergent layer into a sucrose gradient**; yield of cores, %	~10 %	~2 %	~3 %	~19 %	~22 %	Cores recovered from the P38A/T216I and E45A/R132T exhibited CA levels that were similar to those of the corresponding single mutants. Suppressor mutations do not correct the aberrant intrinsic stability of the P38A and E45A mutant capsids [1, 2].
**Disassembly of purified HIV-1 cores**; recovery, % of the total CA released from the cores during incubation	~50 % CA released from the cores	ND[a]	ND	~16 % CA released from the cores	~21 % CA released from the cores	The poor recovery of core-associated CA from the P38A mutant particles precluded its analysis. Cores isolated from E45A and E45A/R132T both exhibited slower uncoating [1, 2].
**Exogenous reverse transcription (RT)**; % wild-type HIV-1 activity	100 %	~106 %	ND	~96 %	ND	No significant defects in exogenous RT activity were observed for the CA mutants. Quantities of active RT enzyme incorporated in the mature particles are similar to those in WT HIV-1 [1].
**Viral RNA content**; % wild-type HIV-1 activity	100 %	~86 %	ND	~102 %	ND	No significant reductions in viral RNA packaging were observed. Quantities of viral RNA incorporated in the mature particles are similar to those in WT HIV-1 [1].

Assay	WT_{CA}	P38A	P38A/T216I	E45A	E45A/R132T	Interpretations, references
**Endogenous RT**; % wild-type HIV-1 activity	100 %	~184 %	ND	~115 %	ND	E45A mutant synthesized quantities of DNA similar to that of wild-type HIV-1. P38A was enhanced by up to twofold relative to WT virions. CA mutations do not markedly impair RNA packaging or formation of a functional ribonucleoprotein complex within the virion [1].
**Turbidity assay**; rate of CA assembly, min	++	+++	+++	+++	+++	E45A and E45A/R132T proteins exhibited accelerated assembly. In reference 33, P38A and P38A/T216I also have high assembly rates-possibly due to high protein concentrations used in the turbidity assays. The effects of mutations on CA assembly *in vitro* were not correlated with biological phenotypes of the corresponding CA mutant viruses [2, 3].
**Assembly competence**; tube formation determined by TEM	++	++	ND	++	ND	"*In vitro* cylinder formation" was reported to be similar for WT, P38A, and E45A CA [4].
**Atomic force microscopy (AFM) analysis**; point stiffness, N/m	Assembled tubes ~0.052 N/m; isolated cores ~0.097 N/m	ND	ND	Assembled tubes ~0.153 N/m; isolated cores ~0.152 N/m	Assembled tubes ~0.146 N/m	E45A mutation elevates capsid stiffness in comparison with that of the WT capsid. The stiffness of E45A/R132T mutant CA assemblies is similar to that of the E45A mutant [5].
Cell-based assays						
**Gag expression in transfected 293T**	Normal	Normal	ND	Normal	ND	Altered Gag expression or stability does not account for any of the phenotypes [6].

Assay	WT_{CA}	P38A	P38A/T216I	E45A	E45A/R132T	Interpretations, references
**Gag processing, particle release, and viral protein packaging**	Normal	Normal	ND	Normal	ND	None of the mutations blocked Gag processing or grossly affected the virion stoichiometries [6].
**Correlative 3D live-cell and cryo-ET approach**; intact cores in HeLa cells	50% enveloped intact virions; no intact cores in the cytoplasm	ND	ND	23% enveloped intact virions; 27% cytoplasmic conical cores	ND	Capsid disassembles quickly after membrane fusion. E45A cores undergo delayed capsid disassembly [7].
**CsA washout assay**; half-life of uncoating	Reference	ND	ND	~1.5 fold increase	Similar to the WT	E45A mutation delays uncoating of the virus. R132T was able to rescue the uncoating kinetics of the E45A mutation to wildtype levels. These alterations are not due to changes in reverse transcription [8, 9].
**Replication in CEM cells**	Replicated	Failed to replicate	Replicated with delay	Failed to replicate	Replicated with delay	Compensatory T216I and R132T mutations partially restore the ability of the corresponding P38A and E45A mutant viruses to replicate in CEM cells [1, 2].
**Single-round reporter assay**; relative infectivity, % CA WT	100 %	~3 %	~36 %	~4 %	~27 %	T216I and R132T markedly enhanced the infectivity of P38A and E45A [1, 2, 4].
**HIV-1 RT in target cells***	Normal	Competent for efficient RT, exhibited unusually rapid kinetics	ND	Severe defects at both early and late stages of RT	ND	P38A was competent for efficient RT, but exhibited unusually rapid kinetics, with DNA synthesis peaking several hours earlier than for WT HIV-1. E45A exhibited severe defects at both early and late stages of RT. The impaired RT probably reflects a specific difference in viral core stability [1].

Assay	WT_{CA}	P38A	P38A/T216I	E45A	E45A/R132T	Interpretations, references
**HIV-1 RT in target cells***; copies of HIV-1 DNA	~60,000<br>Normal	~5,000<br>Impaired RT	~30,000<br>Partially rescued	~65,000<br>Normal RT	~32,000<br>ND	Impaired infectivity of P38A is a result of its reduced RT capacity. T216I partially rescued the impaired RT exhibited by P38A. E45A mutation impairs later stages of infection, after nuclear entry [2].
**Early and late RT***; fold change 15 min post-infection	Reference	ND	ND	2 fold higher than CA WT	ND	More rapid accumulation of early RT products was observed for E45A compared to CA WT. Late RT products did not increase significantly during 155 min time course [10].
**Viral RNA 5-ethynyl uridine (EU) staining kinetics**; beginning of decay, min post-infection	50 min	ND	ND	Staining increased in 15-25 min, followed by decline at 35 min	EU staining resembled CA WT	Capsid of E45A HIV-1 dissociated early after infection. The E45A phenotype was partially reversed with the addition of the mutation of R132T [11].
**Capsid permeability** to antibodies and the fluorescent dye	Little RNA staining; almost no staining of NC	ND	ND	Viral RNA and NC staining	ND	Both the small molecule dye and the anti-NC antibodies could penetrate the E45A cores [11].
**Cell cycle dependence****; relative infectivity in arrested *vs.* control cells, fold change	<0.5 fold reduction	<0.5 fold reduction	<0.5 fold reduction	8 fold reduction	<0.5 fold reduction	P38A and P38A/T216I mutants behaved similar to WT HIV-1. R132T corrects the selective impairment of infection of non-dividing cells associated with the E45A mutant virus [2, 12].
**Sensitivity of viruses to PF74 inhibition in single-cycle infection assays****; IC_{50}, µM	~0.26	Hypersensitive	ND	Resistant	~0.38	E45A exhibited reduced sensitivity to inhibition by PF74 relative to WT HIV-1. P38A was more sensitive, exhibiting a greater reduction of infection at both low and high PF74 concentrations. The

Assay	WT_{CA}	P38A	P38A/T216I	E45A	E45A/R132T	Interpretations, references
						restored sensitivity of the E45A/R132T virus to PF74 suggests that the R132T mutation partially reverses the E45A-induced uncoating defect in target cells [2, 13, 14].
**Extent of PF74 binding**; % of WT	100	~210	ND	~100	ND	P38A bound approximately twice as much of the PF74 as WT HIV-1 particles, potentially contributing to the increased sensitivity to PF74. The compound bound to E45A to an extent comparable to that of the WT, which suggests that altered sensitivity is likely due to increased capsid stability [13].
**The ability to saturate TRIM5 restriction in monkey cells****	Ability to abrogate restriction *in trans*	Impaired	Rescued	Similar to WT	ND	The efficient trans-abrogation of TRIM5 restriction requires particles with a stable capsid. T216I restores the ability of P38A particles to abrogate restriction. Thus, T216I mutation prevents premature disassembly of the P38A mutant core in target cells, thereby relieving its impaired ability to interact with restriction factors [2, 15, 16].
**CPSF6-358 restriction of HIV-1 infection****	Restricted	Retained sensitivity	ND	Resistant in dividing cells, but strongly restricted in the growth-arrested cells	ND	CPSF6-358 interaction with incoming HIV-1 cores impairs productive interactions with uncoating or transport factors [17, 18].

Assay	WT$_{CA}$	P38A	P38A/T216I	E45A	E45A/R132T	Interpretations, references
**Sensitivity to restriction by TRIM-Nup153**	Restricted	Retained sensitivity	ND	Less effective at restricting	ND	Some CA disassembly may be needed for interaction with Nup153 [18, 19].
**HIV-1 sensitivity to Nup85, Nup107, Nup133, Nup153, Nup155, Nup160, and Nup358 depletions****	HIV-1 infection was decreased in cells depleted of Nup155, Nup160, or Nup358	ND	ND	Less sensitive to Nup depletions	ND	E45A HIV-1 mutant interacts inefficiently with the nuclear pore complex; also, its dependence on TNPO3 is different than WT. [17, 19, 20].
**Effect of CypA knockdown or cyclosporine treatment on infectivity in Nup153 depleted cells****	Relatively unaffected	ND	ND	5- to 20-fold increase in infectivity	ND	Perturbation of CypA binding dictated the sensitivity to Nup153 depletion. It appears the amount of CypA bound to the HIV-1 core is able to dictate whether the pre-integration complex undergoes downstream processes requiring Nup153, perhaps by altering the dynamics of uncoating [20].
**HIV-1 dependence on TNPO3 for infection****	Dependent	Partially dependent	ND	Not affected	Partially dependent	Infection by the E45A/R132T double mutant exhibited TNPO3 dependence between those of E45A and the wild-type. The second-site suppressor mutant restores TNPO3 dependence of infection [14, 17, 21].
Structural assessment						
**Nuclear magnetic resonance (NMR)**; chemical shift change, ppm	Reference spectrum	<0.43<br>Dispersed over a wider region (V36, I37, A38, M39, K30-S33, W23-E28, M55, L136, V142)	ND	0.8;<br>Local (A45, G46)	0.3-1<br>Local (A45, G46, I129, T132, W133, L136)	The spectra of all the mutants are very similar to that of WT, demonstrating conservation of the overall global fold. The effects of the P38A may result from subtle changes in the overall structure and from its involvement in the intra-

Assay	WT_{CA}	P38A	P38A/T216I	E45A	E45A/R132T	Interpretations, references
						hexamer interactions. The effects of the E45A and R132T mutations result from the change in chemical nature of the substituted amino acid [2].
**This study**						
In vitro						
**Assembly competence**; cylinder formation as determined by cryo-EM	Long tubes	No tubes	Long tubes	Short tubes and cones	Long tubes	Altered assembly morphologies were reverted to long tubes as the CA WT by the second compensatory mutations.
**Pelleting assay**	++	-	++	+++	++	Second-site mutations reversed assembly efficiencies to levels similar to the *CA WT*.
**CA multimerization assay**	++	+	++	+++	++	Assembly rates of E45A and E45A/R132T were the fastest. P38A formed aggregates very inefficiently; addition of T216I restored the rate of assembly to WT levels.
Cell-based assay						
**CypA-DsRed loss assay**; core stability	Reference	Less stable	Less stable	More stable	More stable than CA WT, but less stable than E45A	E45A/R132T is less stable than E45A, but is more stable than CA WT. P38A and P38A/T216I are slightly different. Collectively, compensatory mutations do not fully correct the intrinsic stability defects imposed by P38A and E45A mutations [22].
Structural assessment						
**X-ray crystallography**	Reference structure	Affected residues: P1, H12, L20, E28, E29, K30, A31, F32,	Additionally: T200, I201, L202, K203,	P1, H12, A45, E128, R132, Q50, D51	P1, H12, A45, E128, T132, Q50, D51	E45 is engaged in repulsive ionic interactions with D51 from the neighboring subunit. E45A

Assay	WT_{CA}	P38A	P38A/T216I	E45A	E45A/R132T	Interpretations, references
		S33, P34, E35, V36, I37, A38, M39, S41, A42, E45, T54, E128, R132, R143, M144, Y145, R162, Q176	A204, L205, G206, P207, G208, M215, I216, A217, Q219, G220, V221			mutation directly relieves electrostatic repulsion, resulting in stabilization of the E45A CA hexamer and the core. In the P38A mutant, the network of interactions around E45 is altered, together with a network of additional residues over 3 neighboring CA subunits (Fig. 1), leading to the “loosening” likely causing the observed destabilization of the P38A CA hexamers and the core. R132T and P216I, are able to partially offset the effect of primary mutations. R132T partially restores the overall net charge of CA, while T216I stabilizes inter-hexamer interactions.

[a] ND – no data
* – these studies show contradictory results
** – the infectivity of P38A and E45A mutant viruses are significantly impaired

Supplementary Table 2. Summary of X-ray data collection and refinement statistics.

	WT_{CPSF6}	WT_{Nup153}	P38A	P38A/T216I	E45A[a]	E45A[b]	E45A/R132T
Data collection							
X-ray source	APS 23 ID-B	ALS 4.2.2	APS 23 ID-B	APS 23 ID-D	APS 23 ID-D	APS 23 ID-B	APS 23 ID-D
Software	XDS	XDS	XDS	XDS	XDS	XDS	XDS
Space group	P6	P6	P6	P6	P6	P6	P6
Unit cell dimensions							
a, *b*, *c* (Å)	92.7 92.7 58.0	92.5 92.5 58.3	92.1 92.1 57.5	92.2 92.2 57.7	87.6 87.6 56.5	92.5 92.5 57.8	92.4 92.4 57.7
α, β, γ (°)	90.0 90.0 120.0	90.0 90.0 120.0	90.0 90.0 120.0	90.0 90.0 120.0	90.0 90.0 120.0	90.0 90.0 120.0	90.0 90.0 120.0
ASU content	1	1	1	1	1	1	1
Wavelength (Å)	1.033203	1.000031	1.0332	1.03319	1.0332	1.0332	1.03319
Resolution range (Å)[a]	47.0–2.5 (2.6–2.5)	36.2–2.4 (2.5–2.4)	46.6–2.4 (2.5–2.4)	46.8–2.6 (2.7–2.6)	37.9–2.5 (2.6–2.5)	46.8–2.2 (2.3–2.2)	46.8–2.0 (2.1–2.0)
R_{merge}	0.063 (0.700)	0.065 (0.689)	0.065 (>1)	0.071 (0.804)	0.144 (0.807)	0.084 (>1)	0.054 (0.992)
R_{meas}	0.069 (0.774)	0.068 (0.723)	0.068 (>1)	0.075 (0.850)	0.162 (0.916)	0.089 (>1)	0.057 (>1)
R_{pim}	0.029 (0.326)	0.021 (0.220)	0.022 (0.344)	0.024 (0.273)	0.073 (0.428)	0.027 (0.417)	0.018 (0.395)
<I/σI>	18.1 (2.7)	24.7 (3.3)	19.6 (2.1)	15.8 (1.7)	9.1 (2.0)	17.6 (1.9)	23.3 (1.9)
$CC_{1/2}$ (%)	99.8 (58.3)	99.9 (90.1)	99.8 (71.6)	99.8 (79.1)	99.2 (64.3)	99.9 (62.8)	100 (58.9)
Completeness (%)	99.9 (100)	97.2 (100)	99.5 (96.7)	99.8 (98.4)	99.3 (95.9)	99.9 (99.2)	99.9 (99.3)
Redundancy	5.6 (5.5)	10.6 (10.8)	10.1 (9.6)	9.8 (9.3)	4.8 (4.4)	11.3 (10.7)	9.8 (7.0)
Mosaicity	0.13	0.17	0.14	0.10	0.23	0.13	0.07
Refinement							
Resolution (Å)	47.0–2.5	36.2–2.4	46.6–2.4	46.8–2.6	34.6–2.5	46.8–2.2	46.8–2.0
No. total reflections	55,693	115,488	110,167	86,275	41,802	160,668	186,695
No. unique reflections	9,956	10,941	10,893	8,751	8,626	14,249	19,126
No. test reflections[b]	598	630	631	521	422	692	918
R_{work} / R_{free}	24.2 / 27.3	23.4 / 27.4	22.8 / 25.6	20.0 / 24.1	20.2 / 25.0	20.3 / 22.3	19.7 / 21.8
No. atoms	1,835	1,806	1,748	1,752	1,801	1,844	1,868
Protein	1,794	1,742	1,702	1,706	1,724	1,732	1,708
Ligand/Ion	10	9	13	12	15	14	15
Water	31	55	33	34	62	98	145
Wilson B-factor ($Å^2$)	47.2	47.5	67.4	77.5	49.6	47.2	40.4
Average B-factors ($Å^2$)	75.2	71.4	87.6	99.9	71.0	63.4	58.1
Protein	75.3	71.7	87.7	100.2	72.2	63.2	58.2

	WT_{CPSF6}	WT_{Nup153}	P38A	P38A/T216I	E45A[a]	E45A[b]	E45A/R132T
Ligand/Ion	96.3	83.1	103.1	118.6	78.7	85.2	66.9
Water	62.4	59.8	76.4	76.5	63.2	62.8	56.8
RMS deviations							
Bond lengths (Å)	0.002	0.002	0.002	0.009	0.002	0.007	0.007
Bond angles (°)	0.53	0.54	0.497	1.27	0.443	1.062	1.106
MolProbity Statistics[c]							
All atom clash score	3.34	3.73	4.98	2.92	4.90	1.43	2.61
Rotamer outliers (%)	0	0	0	0	0	0	0
Cβ deviations>0.25 Å	0	0	0	0	0	0	0
Ramachandran [c]							
Favored region (%)	98	99	99	99	97	98	98
Outliers (%)	0	0	0	0	0	0	0
PDB accession code	6AY9	6AYA	6B2G	6B2H	6B2I	6B2J	6B2K

[a] Values in parentheses are for highest-resolution shell
[b] Random selection
[c] Values obtained from MOLPROBITY

Supplementary Table 3. Interface area, solvation energy gain, and binding energy calculated for various CA structures.

	Intra-hexamer interfaces			Inter-hexamer interfaces								
	CA$_{NTD}$-CA$_{NTD}$ CA$_{NTD}$-CA$_{CTD}$			CA$_{CTD}$-CA$_{CTD}$ 2-fold			CA$_{CTD}$-CA$_{CTD}$ 3-fold			CA$_{NTD}$-CA$_{NTD}$ 3-fold		
**Structure**	*IA*[1]	*ΔG*[2]	*BE*[3]	*IA*	*ΔG*	*BE*	*IA*	*ΔG*	*BE*	*IA*	*ΔG*	*BE*
**WT$_{CPSF6}$**	1,127.2	-13.6	-17.0	421.1	-6.9	-7.7	22.7	-0.4	-0.4	-	-	-
**WT$_{Nup153}$**	1,154.2	-11.2	-16.4	391.4	-7.6	-7.6	29.3	-0.7	-0.7	-	-	-
**P38A**	1,143.8	-13.5	-17.8	442.2	-7.1	-7.1	41.7	-0.8	-0.8	-	-	-
**P38A/T216I**	1,201.4	-13.3	-18.5	467.8	-7.1	-8.0	97.7	-2.1	-2.1	-	-	-
**E45A[a]**	1157.9	-12.4	-16.3	726.2	-8.5	-12.7	236.4	-4.1	-4.1	23.9	0.6	0.6
**E45A[b]**	1,221.6	-14.4	-18.6	409.1	-7.1	-8.0	32.4	-0.6	-0.6	-	-	-
**E45A/R132T**	1,069.8	-13.8	-17.3	421.6	-7.4	-8.2	21.9	-0.4	-0.4	-	-	-
**WT$_{CA}$**	1,118.8	-13.0	-15.6	453.2	-5.8	-6.7	46.3	-1.0	-1.0	-	-	-

[1] *IA*, Å^2 – Interface Area defined as the half sum of the buried surface area

[2] *ΔG*, *kcal/mol* – Solvation Energy gain

[3] *BE*, *kcal/mol* – Binding Energy

Supplementary Table 4. Analysis of distances around residue 45.

Interaction	WT_{CA}	WT_{CPSF6}	WT_{Nup153}	P38A	P38A/T216I	E45A[a]	E45A[b]	E45A/R132T
45/H12	4.6 Å	3.9 Å	3.1 Å	3.6 Å	4.8 Å	5.1 Å	6.3 Å	6.4 Å
45 / H_2O / H12	3.2 / 3.4 Å	N/A	N/A	N/A	N/A	N/A	N/A	N/A
45 / A14	3.7 Å	3.2 Å	3.8 Å	3.4 Å	3.5 Å	3.7 Å	3.3 Å	3.3 Å
45 / I15	4.2 Å	6.0 Å	3.9 Å	3.7 Å	3.8 Å	5.7 Å	5.5 Å	5.4 Å
45 / Q50	3.5 Å	4.4 Å	3.9 Å	3.0 Å	3.4 Å	7.2 Å	6.3 Å	6.2 Å
45 / D51	3.4 Å	6.0 Å	4.1 Å	3.8 Å	3.4 Å	7.3 Å	6.9 Å	6.9 Å
45 / H_2O / D51	N/A	N/A	N/A	2.5 / 2.9 Å	N/A	5.3 / 3.3 Å	4.1 / 3.1 Å	3.6 / 3.6 Å
45 / T54	4.3 Å	7.1 Å	6.5 Å	6.0 Å	4.0 Å	8.1 Å	7.8 Å	7.8 Å
45 / H_2O / T54	3.9 / 4.0 Å	N/A	N/A	2.5 / 3.6 Å	N/A	5.3 / 3.4 Å	5.4 / 3.9 Å	5.4 / 3.9 Å
45 / L111	5.0 Å	3.3 Å	4.5 Å	3.9 Å	5.5 Å	7.6 Å	7.1 Å	7.2 Å
G46 / H_2O / H12	3.7 / 3.4 Å	N/A	N/A	N/A	N/A	4.9 / 3.3 Å	3.9 / 3.2 Å	3.4 / 3.8 Å

Supplementary Table 5. Intra- (CA$_{NTD}$-CA$_{NTD}$ and CA$_{NTD}$-CA$_{CTD}$) hexamer interactions.

Residues participating in hydrogen bonding networks are shown in pink. Interacting residues present in all compared structures are highlighted in blue. All interactions are among domains from neighboring CA subunits.

WT$_{CA}$	WT$_{CPSF6}$	WT$_{Nup153}$	P38A	P38A/T216I	E45A[a]	E45A[b]	E45A/R132T
*CA$_{NTD}$-CA$_{NTD}$ intra-hexamer interface**							
V3 / H12	-	-	V3 / H12	V3 / H12	-	V3 / H12	V3 / H12
-	-	-	V3 / Q13	-	-	V3 / Q13	V3 / Q13
-	-	-	-	-	Q4 / N5	-	-
-	-	-	-	-	Q4 / Q7	-	-
Q4 / V11	Q4 / V11	Q4 / V11	Q4 / V11	Q4 / V11	-	Q4 / V11	Q4 / V11
Q4 / H12	Q4 / H12	Q4 / H12	Q4 / H12	Q4 / H12	-	Q4 / H12	Q4 / H12
Q4 / H2O / H12	-	-	-	-	-	-	-
-	-	-	-	-	-	N5 / V11	-
-	-	-	-	-	-	N5 / L6	-
-	-	-	-	-	-	L6 / L6	-
-	-	-	-	-	-	L6 / Q7	-
-	-	-	-	-	-	Q7 / Q7	-
-	-	-	-	Q7 / Q9	-	Q7 / Q9	-
-	G8 / Q9	-	-	-	-	G8 / Q9	-
R18 / P17	-	-	R18 / P17	R18 / P17	-	R18 / P17	R18 / P17
R18 / R18	R18 / R18	R18 / R18	R18 / R18	R18 / R18	R18 / R18	R18 / R18	R18 / R18
T19 / P17	T19 / P17	T19 / P17	T19 / P17	T19 / P17	T19 / P17	T19 / P17	T19 / P17
-	-	-	-	-	N21 / A22	-	-
-	-	E29 / H2O / K25	-	-	-	-	-
-	-	E29 / H2O / E28	-	-	-	-	-
-	-	K30 / H2O / K25	-	-	-	-	-
K30 / E28	K30 / E28	-	-	K30 / E28	K30 / E28	-	-

WT$_{CA}$	WT$_{CPSF6}$	WT$_{Nup153}$	P38A	P38A/T216I	E45A[a]	E45A[b]	E45A/R132T
-	-	K30 / H2O / E28	-	-	-	-	-
-	-	-	-	-	-	-	K30 / H_2O / T58
-	-	-	-	-	-	-	K30 / H_2O / G60
E35 / N57	-	-	-	E35 / N57	-	E35 / N57	E35 / N57
E35 / T58	-	E35 / T58	-	E35 / T58	E35 / T58	E35 / T58	E35 / T58
E35 / H_2O / T58	E35 / H_2O / T58	-	E35 / H_2O / T58	-	-	E35 / H_2O / T58	E35 / H_2O / T58
-	-	-	-	-	E35 / V59		
E35 / G60	-	-	-	E35 / G60	E35 / G60	E35 / G60	E35 / G60
P38 / H_2O / T54	-	P38 / H_2O / T54	-	-	-	-	-
P38 / N57	A38 / N57	A38 / N57	A38 / N57	A38 / N57	P38 / N57	P38 / N57	P38 / N57
P38 / T58	P38 / T58	P38 / T58	A38 / T58	A38 / T58	P38 / T58	-	-
M39 / V24	M39 / V24	M39 / V24	-	-	M39 / V24	M39 / V24	M39 / V24
M39 / T58	M39 / T58	M39 / T58	M39 / T58	M39 / T58	M39 / T58	M39 / T58	M39 / T58
-	-	S41/ H_2O / Q50	S41/ H_2O / Q50	-	S41/ H_2O / Q50	-	-
S41/ H_2O / T54	-	S41/ H_2O / T54	S41/ H_2O / T54	-	S41/ H_2O / T54	S41/ H_2O / T54	S41/ H_2O / T54
-	-	-	-	-	A42/ H_2O / I15	-	A42/ H_2O / I15
A42 / L20	A42 / L20	A42 / L20	A42 / L20	A42 / L20	A42 / L20	A42 / L20	A42 / L20
A42 / T54	A42 / T54	A42 / T54	A42 / T54	A42 / T54	A42 / T54	A42 / T54	A42 / T54
A42/ H_2O / T54	-	A42/ H_2O / T54	-	-	-	A42/ H_2O / T54	A42/ H_2O / T54
L43 / L20		L43 / L20	-	-	L43 / L20	-	L43 / L20
-		-	-	-	L43 / P17	-	-
E45 / H_2O / H12	E45/H12	E45/H12	E45/H12	-	-	-	-
E45 / A14	E45 / A14	E45 / A14	E45 / A14	E45 / A14	A45 / A14	A45 / A14	A45 / A14
E45 / I15	-	E45 / I15	E45 / I15	E45 / I15	-	-	-
E45 / Q50	-	E45 / Q50	E45 / Q50	E45 / Q50	-	-	-
E45 / D51	-	-	E45 / D51	E45 / D51	-	-	-
-	-	-	E45 / H_2O / D51	-	-	-	-
-	-	-	E45 / H_2O / T54	-	-	-	-

WT_{CA}	WT_{CPSF6}	WT_{Nup153}	P38A	P38A/T216I	E45A[a]	E45A[b]	E45A/R132T
-	E45 / L111	-	E45 / L111	-	-	-	-
G46 / H_2O / H12	-	-	-	-	-	G46 / H_2O / H12	G46 / H_2O / H12
CA_{NTD}-CA_{CTD} intra-hexamer interface							
-	-	-	-	-	R162 / M144	-	R162 / M144
R162 / H_2O / M144	-	-	-	-	-	-	R162 / H_2O / M144
R162 / Y145	R162 / Y145	R162 / Y145	R162 / Y145	R162 / Y145	R162 / Y145	R162 / Y145	R162 / Y145
R162 / H_2O / Y145	-	-	-	-	-	-	R162 / H_2O / Y145
V165 / A64	V165 / A64	V165 / A64	V165 / A64	V165 / A64	V165 / A64	V165 / A64	V165 / A64
D166 / H62	D166 / H62	D166 / H62	D166 / H62	D166 / H62	D166 / H62	D166 / H62	D166 / H62
D166 / H_2O / H62	-	D166 / H_2O / H62	D166 / H_2O / H62	D166 / H_2O / H62	-	D166 / H_2O / H62	D166 / H_2O / H62
D166 / Q63	D166 / Q63	D166 / Q63	D166 / Q63	D166 / Q63	D166 / Q63	D166 / Q63	D166 / Q63
D166 / H_2O / Q63	-	D166 / H_2O / Q63	D166 / H_2O / A63	D166 / H_2O / A63	-	D166 / H_2O / A63	D166 / H_2O / A63
D166 / A64	D166 / A64	D166 / A64	D166 / A64	D166 / A64	D166 / A64	D166 / A64	D166 / A64
D166 / H_2O / A65	-	D166 / H_2O / A65	D166 / H_2O / A65	-	D166 / H_2O / A65	D166 / H_2O / A65	D166 / H_2O / A65
D166 / H_2O / Y145	-	D166 / H_2O / Y145	D166 / H_2O / Y145	-	D166 / H_2O / Y145	D166 / H_2O / Y145	D166 / H_2O / Y145
Y169 / Q63	Y169 / Q63	Y169 / Q63	Y169 / Q63	Y169 / Q63	Y169 / Q63	Y169 / Q63	Y169 / Q63
Y169 / Q67	Y169 / Q67	Y169 / Q67	Y169 / Q67	Y169 / Q67	Y169 / Q67	Y169 / Q67	Y169 / Q67
-	K170 / Q63	K170 / Q63	K170 / Q63	-	K170 / Q63	K170 / Q63	K170 / Q63
R173 / H_2O / L56	R173 / H_2O / L56	R173 / H_2O / L56	-	R173 / H_2O / L56	R173 / H_2O / L56	R173 / H_2O / L56	R173 / H_2O / L56
R173 / N57	R173 / N57	R173 / N57	R173 / N57	R173 / N57	R173 / N57	R173 / N57	R173 / N57
R173 / V59	R173 / V59	R173 / V59	R173 / V59	R173 / V59	R173 / V59	R173 / V59	R173 / V59
R173 / H_2O / V59	R173 / H_2O / V59	R173 / H_2O / V59	-	R173 / H_2O / V59	R173 / H_2O / V59	R173 / H_2O / V59	R173 / H_2O / V59
R173 / E63	R173 / E63	R173 / E63	R173 / E63	R173 / E63	R173 / E63	R173 / E63	R173 / E63

WT$_{CA}$	WT$_{CPSF6}$	WT$_{Nup153}$	P38A	P38A/T216I	E45A[a]	E45A[b]	E45A/R132T
R173 / H_2O / E63	R173 / H_2O / E63	R173 / H_2O / E63	-	R173 / H_2O / E63	R173 / H_2O / E63	R173 / H_2O / E63	R173 / H_2O / E63
-	-	Q179 / H_2O / Q67	Q179 / H_2O / Q67	-	-	-	-
-	-	-	-	-	Q179 / Q67	-	-
-	-	Q179 / K70	-	-	Q179 / K70	-	-
-	-	Q179 / H_2O / K70	-	-	-	-	-
-	-	-	-	-	Q179 / H_2O / N74	-	-
-	-	-	-	-	N182 / Q67	-	-
-	-	-	-	-	N182 / H_2O / K70	-	-
-	-	-	-	-	N183 / H_2O / N74	-	-
T210 / E71	T210 / E71	T210 / E71	T210 / E71	T210 / E71	T210 / E71	T210 / E71	T210 / E71
T210 / E75	-	-	T210 / E75	-	-	-	-
L211 / A64	L211 / A64	L211 / A64	L211 / A64	L211 / A64	L211 / A64	L211 / A64	L211 / A64
L211 / Q67	L211 / Q67	L211 / Q67	L211 / Q67	L211 / Q67	L211 / Q67	L211 / Q67	L211 / Q67
L211 / M68	L211 / M68	L211 / M68	L211 / M68	L211 / M68	L211 / M68	L211 / M68	-
L211 / E71	L211 / E71	L211 / E71	L211 / E71	L211 / E71	L211 / E71	L211 / E71	L211 / E71
E212 / M68	-	E212 / M68	E212 / M68	E212 / M68	-	E212 / M68	E212 / M68
E212 / H_2O / E71	-	-	-	-	-	E212 / H_2O / E71	E212 / H_2O / E71
E212 / K140	-	E212 / K140	E212 / K140	-	E212 / K140	E212 / K140	E212 / K140
-	-	E212 / H_2O / K140	-	-	-	-	-
-	-	E212 / R143	E212 / R143	E212 / R143	-	E212 / R143	E212 / R143
E212 / H_2O / R143	-	-	-	-	E212 / H_2O / R143	-	-
E212 / M144	E212 / M144	E212 / M144	E212 / M144	E212 / M144	E212 / M144	E212 / M144	E212 / M144

WT_{CA}	WT_{CPSF6}	WT_{Nup153}	P38A	P38A/T216I	E45A[a]	E45A[b]	E45A/R132T
M215 / A64	-	-	-	-	M215 / A64	M215 / A64	-
M215 / M68	M215 / M68	M215 / M68	M215 / M68	M215 / M68	M215 / M68	M215 / M68	M215 / M68
-	M215 / M144	-	M215 / M144	M215 / M144	M215 / M144	-	M215 / M144
M215 / Y145	M215 / Y145	M215 / Y145	M215 / Y145	-	M215 / Y145	M215 / Y145	M215 / Y145
T216 / M144	T216 / M144	T216 / M144	T216 / M144	I216 / M144	T216 / M144	T216 / M144	T216 / M144
-	-	-	Q219 / M144	Q219 / M144	Q219 / M144	Q219 / M144	Q219 / M144
-	-	-	-	-	-	Q219 / H_2O / M144	-

*All the residues of β-hairpin are resolved only in the E45A[a] and E45A[b] structures.

Supplementary Table 6. Inter- (2-fold and 3-fold CA_{CTD}-CA_{CTD}) hexamer interactions.

Residues participating in hydrogen bonding networks are shown in pink. Interacting residues present in all compared structures are highlighted in blue. All interactions are among domains from neighboring CA subunits.

WT_{CA}	WT_{CPSF6}	WT_{Nup153}	P38A	P38A/T216I	E45A[a]	E45A[b]	E45A/R132T
CA_{CTD}-CA_{CTD} 2-fold inter-hexamer interface							
-	-	-	-	-	R143 / W184	-	-
-	-	-	-	-	R143 / E187	-	-
-	-	-	-	-	R143 / H_2O / E187	-	-
-	-	-	-	-	R143 / T188	-	-
-	-	-	-	-	S149 / Q192	-	-
-	-	-	-	-	I150 / L189	-	-
L151 / L151	L151 / L151	-	L151 / L151	-	L151 / L151	-	-
L151 / L189	-	-	L151 / L189	L151 / L189	L151 / L189	L151 / L189	L151 / L189
L151 / Q192	L151 / Q192	L151 / Q192	L151 / Q192	L151 / Q192	L151 / Q192	L151 / Q192	L151 / Q192
-	-	-	-	-	L151 / N193	-	-
-	-	-	-	-	E175 / W184	-	-
E175 / H_2O / W184	E175 / H_2O / W184	-	-	-	-	-	-
-	-	-	-	-	E175 / Q192	-	-
Q176 / W184	Q176 / W184	-	-	-	-	-	-
Q176 / H_2O / W184	Q176 / H_2O / W184	-	-	-	-	-	-
-	-	-	-	-	Q176 / H_2O / E187	-	-
-	-	-	-	A177 / W184	A177 / W184	-	-
S178 / E180	S178 / E180	S178 / E180	S178 / E180	S178 / E180	S178 / E180	S178 / E180	S178 / E180
-	S178 / H_2O / E180	-	-	-	-	-	-

E180 / E180	E180 / E180	E180 / E180	E180 / E180	E180 / E180	E180 / E180	E180 / E180	E180 / E180
-	E180 / H_2O / E180	-	-	-	-	-	-
-	-	E180 / V181	-	-	-	E180 / V181	E180 / V181
V181 / W184	V181 / W184	V181 / W184	V181 / W184	V181 / W184	V181 / W184	V181 / W184	V181 / W184
W184 / W184	-	W184 / W184	-	-	-	W184 / W184	W184 / W184
W184 / M185	W184 / M185	W184 / M185	W184 / M185	W184 / M185	W184 / M185	W184 / M185	W184 / M185
CA_{CTD}-CA_{CTD} 3-fold inter-hexamer interface							
-	-	-	-	-	I201 / A204	-	-
-	-	-	-	-	K203 / T216	-	-
-	-	-	-	-	K203 / A217	-	-
-	-	-	-	-	K203 / G220	-	-
-	-	-	-	A204 / A204	-	-	-
A204 / H_2O / A204	-	A204 / H_2O / A204	A204 / H_2O / A204	A204 / H_2O / A204	-	A204 / H_2O / A204	A204 / H_2O / A204
-	-	-	-	-	A204 / L205	-	-
-	-	-	-	-	P207 / E212	-	-
-	-	-	-	-	P207 / E213	-	-
-	-	-	-	-	P207 / T216	-	-
CA_{NTD}-CA_{NTD} 3-fold inter-hexamer interface							
-	-	-	-	-	R82 / H_2O / H_2O / H_2O / R82	-	-

3. Uncropped gel from Supplementary Figure 8c

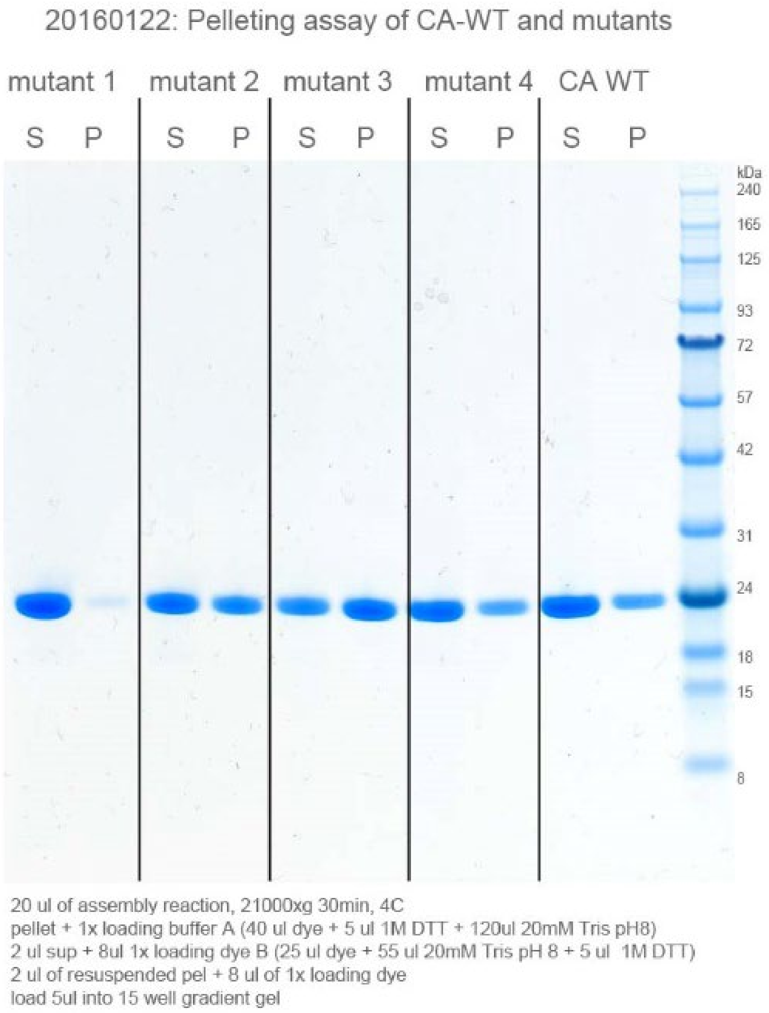

4. Supplementary References

1. Forshey BM, von Schwedler U, Sundquist WI, Aiken C. Formation of a Human Immunodeficiency Virus Type 1 Core of Optimal Stability Is Crucial for Viral Replication. *Journal of Virology* **76**, 5667-5677 (2002).

2. Yang R*, et al.* Second-site suppressors of HIV-1 capsid mutations: restoration of intracellular activities without correction of intrinsic capsid stability defects. *Retrovirology* **9**, 30 (2012).

3. Douglas CC, Thomas D, Lanman J, Prevelige PE, Jr. Investigation of N-terminal domain charged residues on the assembly and stability of HIV-1 CA. *Biochemistry* **43**, 10435-10441 (2004).

4. Ganser-Pornillos BK, von Schwedler UK, Stray KM, Aiken C, Sundquist WI. Assembly properties of the human immunodeficiency virus type 1 CA protein. *J Virol* **78**, 2545-2552 (2004).

5. Ramalho R, Rankovic S, Zhou J, Aiken C, Rousso I. Analysis of the mechanical properties of wild type and hyperstable mutants of the HIV-1 capsid. *Retrovirology* **13**, 17 (2016).

6. von Schwedler UK, Stray KM, Garrus JE, Sundquist WI. Functional Surfaces of the Human Immunodeficiency Virus Type 1 Capsid Protein. *Journal of Virology* **77**, 5439-5450 (2003).

7. Jun S*, et al.* Direct visualization of HIV-1 with correlative live-cell microscopy and cryo-electron tomography. *Structure* **19**, 1573-1581 (2011).

8. Hulme AE, Kelley Z, Okocha EA, Hope TJ. Identification of capsid mutations that alter the rate of HIV-1 uncoating in infected cells. *J Virol* **89**, 643-651 (2015).

9. Ingram Z, Taylor M, Okland G, Martin R, Hulme AE. Characterization of HIV-1 uncoating in human microglial cell lines. *Virol J* **17**, 31 (2020).

10. Ambrose Z*, et al.* Human immunodeficiency virus type 1 capsid mutation N74D alters cyclophilin A dependence and impairs macrophage infection. *J Virol* **86**, 4708-4714 (2012).

11. Xu H*, et al.* Evidence for biphasic uncoating during HIV-1 infection from a novel imaging assay. *Retrovirology* **10**, 70 (2013).

12. Yamashita M, Perez O, Hope TJ, Emerman M. Evidence for direct involvement of the capsid protein in HIV infection of nondividing cells. *PLoS Pathog* **3**, 1502-1510 (2007).

13. Shi J, Zhou J, Shah VB, Aiken C, Whitby K. Small-molecule inhibition of human immunodeficiency virus type 1 infection by virus capsid destabilization. *J Virol* **85**, 542-549 (2011).

14. Shah VB*, et al.* The host proteins transportin SR2/TNPO3 and cyclophilin A exert opposing effects on HIV-1 uncoating. *J Virol* **87**, 422-432 (2013).

15. Forshey BM, Shi J, Aiken C. Structural requirements for recognition of the human immunodeficiency virus type 1 core during host restriction in owl monkey cells. *J Virol* **79**, 869-875 (2005).

16. Zhao G*, et al.* Rhesus TRIM5alpha disrupts the HIV-1 capsid at the inter-hexamer interfaces. *PLoS Pathog* **7**, e1002009 (2011).

17. Lee K, *et al.* Flexible use of nuclear import pathways by HIV-1. *Cell Host Microbe* **7**, 221-233 (2010).

18. Price AJ, *et al.* Host Cofactors and Pharmacologic Ligands Share an Essential Interface in HIV-1 Capsid That Is Lost upon Disassembly. *PLoS Pathog* **10**, e1004459 (2014).

19. Matreyek KA, Yucel SS, Li X, Engelman A. Nucleoporin NUP153 phenylalanine-glycine motifs engage a common binding pocket within the HIV-1 capsid protein to mediate lentiviral infectivity. *PLoS Pathog* **9**, e1003693 (2013).

20. Matreyek KA, Engelman A. The requirement for nucleoporin NUP153 during human immunodeficiency virus type 1 infection is determined by the viral capsid. *J Virol* **85**, 7818-7827 (2011).

21. De Iaco A, Luban J. Inhibition of HIV-1 infection by TNPO3 depletion is determined by capsid and detectable after viral cDNA enters the nucleus. *Retrovirology* **8**, 98 (2011).

22. Francis AC, Marin M, Shi J, Aiken C, Melikyan GB. Time-Resolved Imaging of Single HIV-1 Uncoating In Vitro and in Living Cells. *PLoS Pathog* **12**, e1005709 (2016).
