# Supplementary material for: Multidisciplinary studies with mutated HIV-1 capsid proteins reveal structural mechanisms of lattice stabilization
Source: Nat Commun. 2023 Sep 12;14:5614. doi: 10.1038/s41467-023-41197-7 (PMC10497533; doi:10.1038/s41467-023-41197-7)
Supplement: Supplementary file 2 — Reporting Summary [file 41467_2023_41197_MOESM2_ESM.pdf]

nature portfolio

Corresponding author(s): Sarafianos, Stefan G

Last updated by author(s): Jul 24, 2023

# Reporting Summary

Nature Portfolio wishes to improve the reproducibility of the work that we publish. This form provides structure for consistency and transparency in reporting. For further information on Nature Portfolio policies, see our Editorial Policies and the Editorial Policy Checklist.

## Statistics

For all statistical analyses, confirm that the following items are present in the figure legend, table legend, main text, or Methods section.

| n/a | Confirmed | |
|---|---|---|
| ☐ | ☒ | The exact sample size (*n*) for each experimental group/condition, given as a discrete number and unit of measurement |
| ☐ | ☒ | A statement on whether measurements were taken from distinct samples or whether the same sample was measured repeatedly |
| ☒ | ☐ | The statistical test(s) used AND whether they are one- or two-sided<br>*Only common tests should be described solely by name; describe more complex techniques in the Methods section.* |
| ☒ | ☐ | A description of all covariates tested |
| ☒ | ☐ | A description of any assumptions or corrections, such as tests of normality and adjustment for multiple comparisons |
| ☐ | ☒ | A full description of the statistical parameters including central tendency (e.g. means) or other basic estimates (e.g. regression coefficient) AND variation (e.g. standard deviation) or associated estimates of uncertainty (e.g. confidence intervals) |
| ☒ | ☐ | For null hypothesis testing, the test statistic (e.g. *F*, *t*, *r*) with confidence intervals, effect sizes, degrees of freedom and *P* value noted<br>*Give P values as exact values whenever suitable.* |
| ☒ | ☐ | For Bayesian analysis, information on the choice of priors and Markov chain Monte Carlo settings |
| ☒ | ☐ | For hierarchical and complex designs, identification of the appropriate level for tests and full reporting of outcomes |
| ☒ | ☐ | Estimates of effect sizes (e.g. Cohen's *d*, Pearson's *r*), indicating how they were calculated |

*Our web collection on statistics for biologists contains articles on many of the points above.*

## Software and code

Policy information about availability of computer code

| | |
|---|---|
| Data collection | XDS (2016) software package was used for X-ray diffraction data processing. For SAD data, CRANK-2 (2.0.111) was used to determine phases and initial structures. Phaser (2.7.17) was used for molecular replacement. Several rounds of iterative model building and refinement were carried out using Coot (0.8.8) and PHENIX (1.11.1-2575_1692), REFMAC (5.8.0155), or PDBREDO (6.24), respectively. |
| Data analysis | X-ray diffraction data were analyzed for space group and twinning using POINTLESS (1.10.21) or XTRIAGE (1.11.1-2575_1692). MolProbity server (4.5.2) was used for crystal structure validation. PISA (1.5.0) was used to analyze of buried surface area. PyMOL was used to make figures (1.7.6.7). VMD (1.9.4), NAMD (2.12 and 2.14), APBS (3.1.3) were used in molecular modeling calculations and analysis. |

For manuscripts utilizing custom algorithms or software that are central to the research but not yet described in published literature, software must be made available to editors and reviewers. We strongly encourage code deposition in a community repository (e.g. GitHub). See the Nature Portfolio guidelines for submitting code & software for further information.

## Data

Policy information about availability of data

All manuscripts must include a data availability statement. This statement should provide the following information, where applicable:

- Accession codes, unique identifiers, or web links for publicly available datasets
- A description of any restrictions on data availability
- For clinical datasets or third party data, please ensure that the statement adheres to our policy

The authors declare that the data supporting the findings of this study are available within the paper and its Supplementary Information file. The source data

underlying Figure 4 and Supplementary Figures 8c, 9b-c, 13a-c, 14a-c, and 18 are provided as a Source Data file. X-ray crystal structure coordinates and structure factor data have been deposited into the RCSB Protein Data Bank (PDB) under accession codes
6AY9 [http://doi.org/10.2210/pdb6AY9/pdb] (WT CA/CPSF6),
6AYA [http://doi.org/10.2210/pdb6AYA/pdb] (WT CA/Nup153),
6B2G [http://doi.org/10.2210/pdb6B2G/pdb] (P38A CA),
6B2H [http://doi.org/10.2210/pdb6B2H/pdb] (P38A/T216I CA),
6B2I [http://doi.org/10.2210/pdb6B2I/pdb] (E45Aa CA),
6B2J [http://doi.org/10.2210/pdb6B2J/pdb] (E45Ab CA),
6B2K [http://doi.org/10.2210/pdb6B2K/pdb] (E45A/R132T CA). The manuscript also refers to existing crystal structures 4XFX [http://doi.org/10.2210/pdb4XFX/pdb] (WT CA) and 4XFZ [http://doi.org/10.2210/pdb4XFZ/pdb] (WT CA/PF74). The molecular dynamics datasets generated and analyzed during the current study are available in the Zenodo repository [https://doi.org/10.5281/zenodo.8180089]. Reprints and permissions information is available at www.nature.com/reprints. Readers are welcome to comment on the online version of this article at www.nature.com/nature. Correspondence should be addressed to SGS (stefanos.sarafianos@emory.edu).

## Human research participants

Policy information about studies involving human research participants and Sex and Gender in Research.

| | |
|---|---|
| Reporting on sex and gender | Does not apply |
| Population characteristics | Does not apply |
| Recruitment | Does not apply |
| Ethics oversight | Does not apply |

Note that full information on the approval of the study protocol must also be provided in the manuscript.

# Field-specific reporting

Please select the one below that is the best fit for your research. If you are not sure, read the appropriate sections before making your selection.

☒ Life sciences ☐ Behavioural & social sciences ☐ Ecological, evolutionary & environmental sciences

For a reference copy of the document with all sections, see nature.com/documents/nr-reporting-summary-flat.pdf

# Life sciences study design

All studies must disclose on these points even when the disclosure is negative.

| | |
|---|---|
| Sample size | We selected at least 4 neighboring fields of view per experiment for fluorescence microscopy imaging in each of 4 biological experiments. For MD simulations of in silico thermal stability of 2x3x3 hexameric CA lattices, we used n=32 dimer interfaces from each 2x3x3 hexameric lattice simulation of WT, E45Aa, E45Ab, and E45A/R132T lattices. For MD simulations of ion and water transfer rates of CA hexamers, rates were computed from intervals of 1,000 frames, yielding n=12 inward and outward rate measurements per simulation, over which means and standard errors were computed. Analysis of van der Waals interaction distributions considered the interaction energies of sets of atoms, situated at inter-hexameric interfaces and identified via distance cutoff, over which the statistics were computed. For wild type, n=6,151 atoms; for P38A, n=4,074 atoms; for P38AT16I, n=4,585 atoms; for E45Aa, n=3,362 atoms; for E45Ab, n=4,287 atoms; for E45AR132T, n=3,597 atoms. |
| Data exclusions | No data were excluded from the analyses. |
| Replication | We ensured reproducibility of our results using biological and technical replicates/experiments. Unless otherwise indicated, results/data were reproduced by at least 3 independent biological experiments. |
| Randomization | Design and development of experimental strategies was in most cases done by the same person who performed experiments; therefore, randomization was not possible. Fluorescence microscopy data collection was performed using randomly selected neighboring fields of view, and software-based analysis of fluorescence intensities was randomized. |
| Blinding | Blinded data collection was not possible, since the same person carrying out the data collection was also responsible for the experimental design. |

# Reporting for specific materials, systems and methods

We require information from authors about some types of materials, experimental systems and methods used in many studies. Here, indicate whether each material, system or method listed is relevant to your study. If you are not sure if a list item applies to your research, read the appropriate section before selecting a response.

## Materials & experimental systems

| n/a | Involved in the study |
|---|---|
| x | Antibodies |
|  | x Eukaryotic cell lines |
| x | Palaeontology and archaeology |
| x | Animals and other organisms |
| x | Clinical data |
| x | Dual use research of concern |

## Methods

| n/a | Involved in the study |
|---|---|
| x | ChIP-seq |
| x | Flow cytometry |
| x | MRI-based neuroimaging |

# Eukaryotic cell lines

Policy information about cell lines and Sex and Gender in Research

| | |
|---|---|
| Cell line source(s) | 293T cells were from ATCC. |
| Authentication | The 293T cell lines were authenticated by ATCC. |
| Mycoplasma contamination | The 293T cell lines were tested for mycoplasma contamination and found to be negative. |
| Commonly misidentified lines (See ICLAC register) | No commonly misidentified cell lines were used in this study. |
